# Non-covalent reconfigurable microgel colloidosomes with a well-defined bilayer shell†

**DOI:** 10.1039/d2sc01082h

**Published:** 2022-04-26

**Authors:** Xin Guan, Yang Liu, Zhili Wan, Ying-Lung Steve Tse, To Ngai

**Affiliations:** Department of Chemistry, The Chinese University of Hong Kong Shatin N. T. Hong Kong China stevetse@cuhk.edu.hk tongai@cuhk.edu.hk zhiliwan@scut.edu.cn; School of Food Science and Technology, South China University of Technology Guangzhou 510640 China

## Abstract

Microgels are extremely interfacially active and are widely used to stabilize emulsions. However, they are commonly used to stabilize oil-in-water emulsions due to their intrinsic hydrophilicity and initially dispersed in water. In addition, there have been no attempts to control microgel structural layers that are formed at the interface and as a result it limits applications of microgel in advanced materials. Here, we show that by introducing octanol into poly(*N*-isopropylacrylamide-*co*-methacrylic acid) (PNIPAM-*co*-MAA) microgels, octanol-swollen microgels can rapidly diffuse from the initially dispersed oil phase onto the water droplet surface. This facilitates the formation of microgel-laden interfacial layers with strong elastic responses and also generates stable inverse water-in-oil Pickering emulsions. These emulsions can be used as templates to produce microgel colloidosomes, herein termed ‘microgelsomes’, with shells that can be fine-tuned from a particle monolayer to a well-defined bilayer. The microgelsomes can then be used to encapsulate and/or anchor nanoparticles, proteins, vitamin C, bio-based nanocrystals or enzymes. Moreover, the programmed release of these substances can be achieved by using ethanol as a trigger to mediate shell permeability. Thus, these reconfigurable microgelsomes with a microgel-bilayer shell can respond to external stimuli and demonstrate tailored properties, which offers novel insights into microgels and promise wider application of Pickering emulsions stabilized by soft colloids.

## Abstract 2

Inverse W/O Pickering emulsions and reconfigurable microgelsomes with a well-defined bilayer structure are prepared from octanol-swollen PNIPAM-*co*-MAA microgels and the combination of binary microgels, which promise wider application of soft colloids.

## Graphical abstract

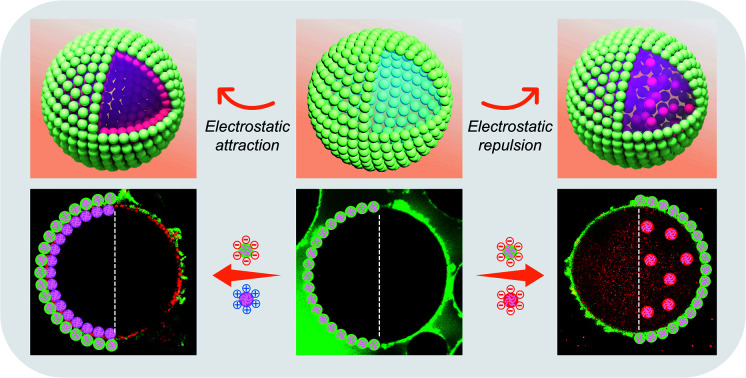

## Introduction

In contrast to traditional Pickering emulsions stabilized by rigid particles, microgels as soft colloids are regarded as more desirable emulsifiers because they can stretch and deform at the oil–water interface.^1–5^ Poly(*N*-isopropylacrylamide) (PNIPAM)-based microgels are one of the most investigated types of soft colloidal particles, as their deformability and stimuli-responsiveness endow them with unique interfacial behaviours, which vary according to their functional groups and microstructure.^6–9^ The different functional groups in microgels also endow as-prepared microgel-stabilized emulsions with multiple functions.^10,11^ For example, poly(*N*-isopropylacrylamide-*co*-methacrylic acid) (PNIPAM-*co*-MAA) microgels bear carboxyl groups (–COOH) that can stabilize emulsions in response to both thermal and pH changes. In addition, the size and cross-linking density of microgels substantially affect their deformability at the oil–water interface, resulting in various interfacial activities and adsorption behaviours.^12–14^ Specifically, microgels with a large size or higher cross-linking density normally exhibit poor particle adsorption and a low surface-coverage ratio. Due to their intrinsic hydrophilicity, PNIPAM-based microgels have typically been used to stabilize oil-in-water (O/W) emulsions.^15,16^ Very recently, Stock *et al.* summarized the development and stabilization mechanism of microgels at the oil-water interface in water-in-oil (W/O) emulsions.^17^ Whereas very few studies have focused on the preparation of W/O emulsions using inherently hydrophilic microgels as the sole emulsifier, because the requirement of oil with high polarity strongly limits their applications.^18,19^

Normally, the intermediate wettability of particles and an external supply of energy (such as homogenization or ultrasonication) are necessary to facilitate particle adsorption at the fluid–fluid interface, consequently yielding a significant increase in the surface coverage of particles and resulting in better emulsion stability.^20,21^ Nevertheless, the strong input of energy or high shear rate may lead to the flocculation of prepared emulsions.^22^ Without using an external force, the spontaneous adsorption of colloidal particles at the fluid interface is an alternative approach.^23–25^ Compared to rigid particles, even though PNIPAM-based microgels are softer and more interfacially active, it remains challenging so far to achieve high packing fractions of such microgels at the an oil–water interface *via* diffusion.

The use of binary particles in emulsion systems has been found to be an effective way to improve the stability of Pickering emulsions.^26–28^ Moreover, Pickering emulsions with an interfacial bilayer structure based on the electrostatic interaction of two stabilizers with opposite charges have recently been developed.^29–31^ Such bilayer structures in Pickering emulsions not only enhance their stability, but also enable microencapsulation and the controlled release of active ingredients. By replacing one type of particle with a polyelectrolyte, similar electrostatic attraction can also be applied to construct colloidosomes.^32,33^ Traditionally, self-assembled colloidal particles can be fixed at the oil–water interface *via* polyelectrolyte complexation. The reversible physical crosslinking between particles and polymers enables the fabrication of robust colloidosomes with flexible and deformable interfacial structures.^34^ Thus far, most reported colloidosomes consist of hard colloids with a monolayer structure.^35^ The build-up of oriented particle bilayer as the shell of colloidosomes was rarely reported.^36^

Even though colloidosomes have been successfully prepared by microgels, they always templated from O/W emulsions with irreversible chemical crosslinking or time-consuming preparation procedures, as most microgels are intrinsically hydrophilic and thus initially dispersed in water.^37–40^ To the best of our knowledge, there have been no attempts to fabricate colloidosomes with tuneable properties from binary microgels with complementary properties in two immiscible liquids. Furthermore, the interactions between binary microgels adsorbed at the oil–water interface and the effects of their self-assembled structure at the oil–water interface on emulsion properties remain largely unexplored.

Herein, we proposed a simple method to prepare inverse W/O Pickering emulsions and colloidosomes composed of binary microgels (termed microgelsomes). We found that PNIPAM-*co*-MAA microgels were *in situ* hydrophobically modified with highly improved interfacial activity in the oil phase by introducing an intermediate concentration of octanol into the special biphasic system (hydrocarbon oil + octanol and water), thus facilitating the formation of inverse W/O Pickering emulsions. After introducing another hydrophilic microgel with complementary properties in the water phase, the fabrication of microgelsomes laden with a bilayer structure can be achieved by spontaneous self-assembly of the binary microgels at the interface, which is driven by differentiated particle diffusion and later interparticle attraction.

In this work, we offer an in-depth understanding of the interaction between octanol and microgels, and the interaction between complementary binary microgels at the fluid–fluid interface, aiming at the design, optimization, and formation of inverse Pickering W/O emulsions solely stabilized by microgels and as-templated microgelsomes with unique self-assembled bilayer structures for potential applications, such as protection and programmed release of various substances under specific conditions.

## Results and discussion

### Inverse W/O Pickering emulsion formation

**Fig. 1:**
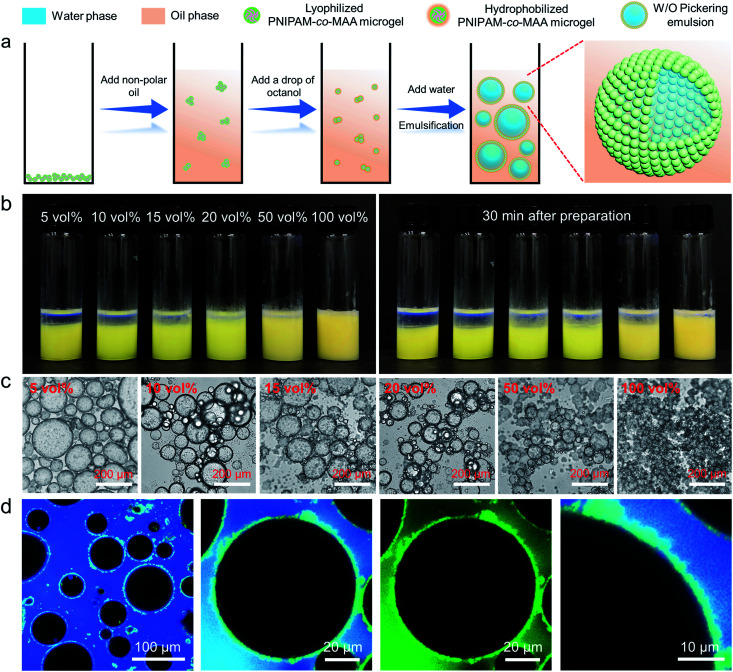
(a) Schematic illustration of the preparation process and stabilization mechanism of inverse W/O Pickering emulsions stabilized by octanol-swollen PNIPAM-*co*-MAA microgels. (b) Photographs and (c) optical microscopy images of inverse W/O Pickering emulsions (W : O = 1 : 1, v/v) stabilized by 1 wt% PNIPAM-*co*-MAA microgels in the oil phase and containing different concentrations of octanol. (d) CLSM images of the inverse W/O Pickering emulsion containing 20 vol% octanol.

It has been reported that PNIPAM microgels can form stable W/O emulsions when a fatty alcohol, such as octanol, was used as the oil phase.^19^ We hypothesize that octanol-swollen microgels could be modified *in situ* to increase hydrophobicity and improve interfacial affinity. As shown in Fig. 1a, to investigate the effect of octanol on microgel properties and emulsification capability, we first dispersed lyophilized microgels into an apolar oil phase such as toluene or dodecane, followed by adding a small amount of octanol. Interestingly, with the presence of octanol, stable W/O emulsions could be formed even with concentrations of octanol as low as 5 vol% (Fig. 1b). As it was reported that the surface properties of microgels can be tuned by the dispersion medium,^41^ we hypothesize that the OH heads of octanol molecules first form hydrogen bonds with multiple electronegative (such as oxygen and nitrogen) atoms of PNIPAM-*co*-MAA microgels. The hydrophobic tails of octanol molecules then surround the microgels, increase their overall hydrophobicity and facilitate their attachment to the oil–water interface, thus forming stable W/O emulsions. The photographs and optical microscopy images (Fig. 1b and c) clearly show that as the concentration of octanol was increased, the emulsions displayed less sedimentation with smaller droplet sizes. CLSM images (Fig. 1d) further prove that microgels were adsorbed at the oil–water interface and formed a dense particle layer around water droplets. It was evident that microgels did not disperse into the water phase, which demonstrate that the octanol-swollen microgels were hydrophobic and preferred to be tightly adsorbed at the interface, instead of transferring from the oil phase into the water phase.

We further investigated the effect of octanol concentrations in terms of microgels in different mediums on the preparation of emulsions. The results indicate that the emulsion type was highly dependent on both the initial dispersed phase of microgels and the octanol concentration (Fig. S1†). In general, when microgels were initially filled with water molecules, the emulsion type began shifting from O/W to W/O at 20 vol% octanol (Fig. S2†). In this case, the dominant emulsion type was hard to define, and a stable W/O emulsion could only be formed by the addition of more than 80 vol% octanol. However, when microgels were pre-swollen by octanol in the oil phase, a stable W/O emulsion could be formed by the addition of only 5 vol% octanol.

### Interaction between octanol and microgels

**Fig. 2:**
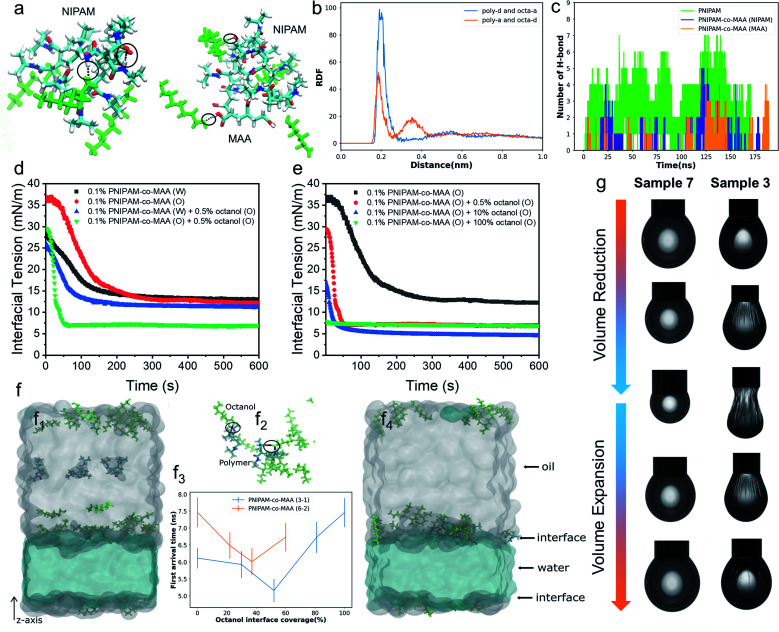
(a) Snapshots of PNIPAM (12) and PNIPAM-*co*-MAA (12-4) systems in molecular dynamics (MD) simulation. The hydrogen bonds between the polymers and octanol (green colour) are represented by black lines and circles. O, N, H and C atoms in the polymers are coloured red, blue, white and cyan, respectively. (b) Radial distribution functions (RDFs) between hydrogen-bond acceptors and donors. (c) Total number of hydrogen bonds between the PNIPAM polymer and octanol, and between the NIPAM subunits or MAA groups of the PNIPAM-*co*-MAA polymer and octanol. Dynamic interfacial tension of the water–toluene interface for (d) 0.1 wt% PNIPAM-*co*-MAA microgels in different phases in the presence or absence of 0.5 vol% octanol and (e) 0.1 wt% PNIPAM-*co*-MAA microgels in the oil phase in the presence of different concentrations of octanol. (f) Dynamics of the PNIPAM-*co*-MAA polymer near the oil–water interface. The starting and the last frames of the simulation with the PNIPAM-*co*-MAA (3-1) polymer near and at the water–oil interface are shown in panels (f_1_) and (f_4_), respectively. (f_2_) Hydrogen bonds (as black lines and circles) formed between PNIPAM-*co*-MAA (3-1) polymers and octanol close to the interface. (f_3_) First arrival time of the polymer from the centre of the oil region to the interface. (g) Appearance of pendant drops at adsorption–desorption equilibrium during the volume reduction and expansion processes. Interfacial jamming and obvious wrinkles were appeared on the drop surface of Sample 3. Sample 3: deionized water – 0.1 wt% PNIPAM-*co*-MAA microgels (oil) + 0.5 vol% octanol (oil); Sample 7: 0.1 wt% PNIPAM-*co*-MAA microgels (water) – 0.5 vol% octanol (oil).

To further elucidate the hydrogen-bonding interaction between PNIPAM-*co*-MAA microgels and octanol, we carried out molecular dynamics (MD) simulations using OpenMM^42,43^ (See “Simulation Details” in the ESI†). Two types of polymers, PNIPAM-*co*-MAA (12-4) and PNIPAM (12), were chosen as the model molecules. PNIPAM-*co*-MAA (12-4) represents a polymer composed of 12 NIPAM molecules and 4 MAA molecules. PNIPAM (12) represents a polymer composed of 12 NIPAM molecules. In the simulations, only one polymer was solvated in the toluene and octanol mixed solution. In Fig. 2a, the hydrogen bonds formed between the polymers, and between polymers and octanol, are shown as black lines. The radial distribution functions (RDFs) between hydrogen-bond acceptors and donors are shown in Fig. 2b. In both the polymers and octanol, oxygen and nitrogen atoms were regarded as the acceptors, and hydrogen atoms connected to the oxygen and nitrogen atoms were regarded as donors. The hydrogen bonds between the polymer donors and octanol acceptors (poly-d and octa-a), and between polymer acceptors and octanol donors (poly-a and octa-d) were considered separately. In both cases, the positions of the first valleys are close to 0.3 nm (Fig. 2b). Thus, we considered that there is a hydrogen bond if the distance between the donor and acceptor is smaller than 0.3 nm. The numbers of hydrogen bonds formed between the polymers and octanol are quantified in Fig. 2c.

The simulation results clearly indicate that hydrogen bonds were formed at several locations between both subunits (NIPAM and MAA) of polymers and octanol. The number of hydrogen bonds increased as the system size increased, so that the effects from hydrogen bonding should be significantly amplified in the experiment compared to the simulation. Furthermore, gas chromatography and water contact angle analysis were performed to verify the interaction between octanol and microgels as well as the successful hydrophobic modification of octanol-swollen microgels (Fig. S3 and S4†). Therefore, it was considered that octanol-swollen microgels are hydrophobic and more effective to promote inverse W/O emulsion formation. Based on these simulation and experimental results, we speculate that although PNIPAM-*co*-MAA microgels have a great affinity and strong interaction with octanol, microgels that are initially dispersed in a water phase can only absorb octanol from the oil–water interface during emulsification, which results in a much slower modification process. In contrast, the interaction between microgels and octanol in the oil phase is stronger and faster, because of the greatly increased contact area and the sufficient absorption time before emulsification.

### Spontaneous self-assembly of microgels at the interface

To gain more insight into the interfacial assembly process, the dynamic adsorption and dilatational rheology of microgels at the interface were measured. As shown in Fig. 2d, the medium in which PNIPAM-*co*-MAA microgels were dispersed and the presence of octanol strongly affected the microgels' adsorption kinetics. In the absence of octanol, the PNIPAM-*co*-MAA microgels in the oil phase demonstrated slower initial adsorption than those pre-dispersed in water, suggesting that the lower diffusion rate of microgels from oil to the interface may be due to the presence of larger microgel aggregates (Fig. S5†). Once these microgels had diffused to the interface, a faster decrease in interfacial tension was observed, and the microgels exhibited similar equilibrated interfacial tension values to microgels pre-dispersed in water. This means that the dispersion medium (water or oil) does not markedly affect the equilibrated density and composition of the microgel-laden interfacial layers, but does strongly affect the kinetics of the adsorption process. After the addition of relatively more polar octanol to the oil, the PNIPAM-*co*-MAA microgels pre-dispersed in water displayed similar interfacial tension decay curves, although the presence of a low concentration (0.5 vol%) of octanol led to a decrease in equilibrium interfacial tension, which can be attributed to the formation of a polar octanol–water interface (Fig. S6†). In contrast, the addition of octanol dramatically affected the interfacial adsorption of the microgels pre-dispersed in the oil phase. As can be seen, the octanol-swollen PNIPAM-*co*-MAA microgels showed a rapidly decreased interfacial tension to approximately 7 mN m^−1^, and an equilibrium adsorption was reached within only 60 s, indicating a significantly enhanced interfacial activity.

We next investigated the influence of octanol concentration on the interfacial assembly of PNIPAM-*co*-MAA microgels. From Fig. S6,† it can be clearly seen that the increasing octanol concentration led to a decrease in the interfacial tension at the water–toluene interface, due to the solvation effect of octanol. We found that a low concentration of octanol (0.5 vol%) greatly improved the interfacial activity of PNIPAM-*co*-MAA microgels in the oil phase (Fig. 2e), whereas an increased concentration (10 vol%) caused only a slight decrease in the equilibrium interfacial tension. Moreover, when the microgels were dispersed in pure octanol solution, they did not significantly affect the decay of interfacial tension as a function of time. This is supported by the result (Fig. S7†) that shows the lyophilized microgel aggregates disintegrated in octanol and became a single microgel dispersion. Therefore, they mostly prefer to remain in the bulk oil solution due to the high polarity of the octanol–water interface with a very low interfacial tension (8 mN m^−1^, Fig. S6†). Accordingly, the surface-coverage ratio of the microgels on water drops sharply decreased, without wrinkles appearing on drop surfaces (Fig. S8 and Movie S1†).

To thoroughly investigate the microgel adsorption kinetics in the presence of octanol, we set up another MD simulation to study the dynamics of the polymers at the oil–water interface at different octanol concentrations (Fig. 2f). The polymer PNIPAM-*co*-MAA with two different sizes, (3 NIPAM and 1 MAA) & (6 NIPAM and 2 MAA), were simulated using different interface coverage degree of octanol, as shown in Fig. 2f_3_. In the simulations of both polymer chains, the first arrival time, the time at which the polymer chain first arrives at the interface, initially decreased and then increased with increasing octanol interface coverage. During the 15 ns in the simulations, most of the octanol molecules gathered on the oil side of the interface, and when the polymer molecules diffused close to the interfacial region, the octanol molecules formed hydrogen bonds with the polymer molecules, thereby accelerating the approach of polymer molecules to the interface (shown in Fig. 2f_2_). When the interfacial region was saturated with octanol, the steric hindrance from the octanol, in contrast, slowed down the polymer approach. In addition, the general trends in the first arrival times for both polymers with different chain lengths were similar (Fig. 2f_3_). These trends were consistent with the experimental results in Fig. 2e, in which the interfacial tension decreasing rate was higher at relatively low octanol concentrations. When toluene was completely replaced with octanol, the interfacial tension barely changed throughout the experiment. In addition, the first arrival time for PNIPAM-*co*-MAA (6-2) was found to be longer than that of PNIPAM-*co*-MAA (3-1), as the longer polymer diffused more slowly. It is worth noting that a lower octanol concentration also had a greater effect on the adsorption of this longer polymer (PNIPAM-*co*-MAA (6-2)). Hence, it is plausible that the much larger PNIPAM-based microgel used in the experiment would take much longer (possibly up to seconds) to reach the interface and thereby affect the interfacial tension (Fig. 2e). These results suggest that microgel adsorption is not necessarily enhanced more by higher concentrations of octanol.

Fig. 2g shows the appearance of pendant drops laden with a microgel monolayer during the deformation process of surface compression and expansion. It can be clearly seen that unusual wrinkles were formed over the entire surface of the drop (Sample 3) when a large compression was applied (Movie S2†). These wrinkles demonstrate a clear manifestation of the strong rheological responses of a highly elastic monolayer assembled by PNIPAM-*co*-MAA microgels from the oil phase; these were much stronger responses than those that have been previously reported.^15,44^ This phenomenon was explained as the high surface coverage of octanol-swollen microgels led to the spontaneous buckling and jamming of the adsorption monolayer during compression. In contrast, even though water-swollen microgels are able to adsorb at the oil-water interface, very limited parts of microgels can deform and flatten at the interface because of their intrinsic hydrophilicity.^6,45,46^ In addition, the electrostatic repulsion between like-charged microgels at the interface may lead to their sparse distribution. This accounts for the fact that no wrinkles were formed on the drop surface of Sample 7 (Movie S3†). We conjecture that octanol-swollen microgels with a larger water contact angle may better embed at the interface and interconnect with each other than those with small water contact angles. The presence of octanol may replace the charge stabilizing ions from microgels by competitive adsorption,^23,47,48^ thus weakening the in-plane electrostatic repulsion and reducing the gap between adjacent microgels. Additionally, the octanol-swollen microgel rearranged and densely packed on the drop surface, which led to strong attractive capillary interactions during the deformation process. This is also supported by the corresponding Lissajous plots which show that compared to the water-swollen microgel covered surface, the surface occupied by octanol-swollen microgels exhibits strain hardening with a pronounced elastic response in compression even at small deformations (4%) (Fig. S9a and c†).

### *In situ* formation of microgelsomes with an interfacial bilayer structure

**Fig. 3:**
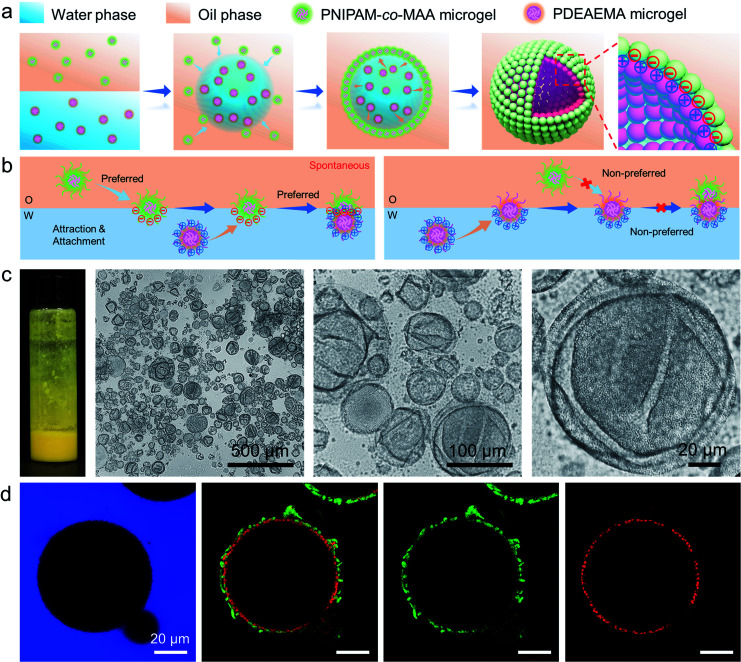
(a) Schematic illustration of the formation process and interfacial bilayer structure of the microgelsome constructed from oppositely charged binary microgels. (b) Proposed stabilization mechanism of the microgel bilayer at the interface, with oppositely charged microgels dispersed in different phases. (c) Photographs, optical microscopy images and (d) CLSM images of non-covalent microgelsomes during solvent evaporation, stabilized by 0.5 wt% PNIPAM-*co*-MAA microgels (green) in the oil phase and 0.5 wt% PDEAEMA microgels (red) in the water phase. The microgelsomes composed of a microgel bilayer structure with numerous wrinkles appeared on the surface.

Recently, emulsions constructed from binary particles have gained traction due to their unique interfacial structures. This interest stems from the fact that the combined assembly of various particles at the oil–water interface may afford as-prepared emulsions with improved stabilities and novel functionalities. Thus, we simultaneously introduced two types of microgels into the biphasic system—poly(2-(diethylamino)ethyl methacrylate) (PDEAEMA) microgel into the water phase and PNIPAM-*co*-MAA microgels into the oil phase — to explore their interaction and combined effect on the dynamics and structures of interfaces and emulsions. The formation process of microgelsomes with an interfacial bilayer is shown in Fig. 3a. Surprisingly, we found that capsule-like droplets were formed after simple vortexing. Wrinkles were clearly visible on the droplet surface during solvent evaporation (Fig. 3c). The corresponding CLSM images clearly indicate that binary microgels led to a W/O type emulsion, and the droplet surface was laden with two layers of microgels (Fig. 3d). In contrast to the W/O emulsion solely stabilized by PNIPAM-*co*-MAA microgels (Fig. 1d), microgelsomes with an interfacial bilayer structure can be constructed *in situ* from oppositely charged PNIPAM-*co*-MAA microgels as their outer layer and PDEAEMA microgels as their inner layer. The possible stabilization mechanism of the microgel bilayer is illustrated in Fig. 3b. We suppose that octanol-swollen PNIPAM-*co*-MAA microgels are first adsorbed at the interface during emulsification, which generates the observed inverse W/O Pickering emulsions. The preferential adsorption of PNIPAM-*co*-MAA microgels (rather than PDEAEMA microgels) at the interface is attributable to the higher hydrophilicity of the latter microgels, which decreases their affinity to the interface (Fig. S10 and S11†). It was considered that these two types of microgels are oppositely charged under neutral conditions because of complementary functional groups, and thus once the PNIPAM-*co*-MAA microgels have occupied the interface, the electrostatic attraction between the oppositely charged microgels drives the diffusion of positively charged PDEAEMA microgels onto the interface. Such a bilayer structure can also be achieved by using other combinations of oppositely changed particles, such as positively charged PS-NH_4_^+^ latex particles and negatively charged PNIPAM-*co*-MAA microgels (Fig. S12†). These results suggest that electrostatic-driven interfacial assembly of binary microgels from both oil and water phases ultimately leads to the formation of the unique interfacial bilayer structure.

### Electrostatic interaction between charged microgels at different pH values

**Fig. 4:**
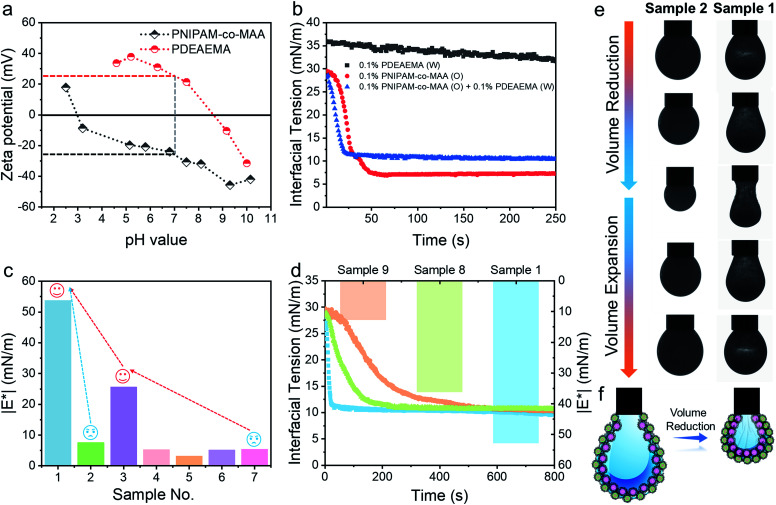
(a) ζ Potential measurements of PNIPAM-*co*-MAA microgels and PDEAEMA microgels in water as a function of pH value. (b) Dynamic interfacial tension of the water–toluene interface for a single microgel or binary microgels system in the presence of 0.5 vol% octanol. (c) Complex moduli (|E*|) of the pendant drop surface formed under different conditions. (d) Dynamic interfacial tension of the water–toluene interface and |E*| of the pendant drop surface for different concentrations of binary microgels dispersed in immiscible phases. (e) Appearance of pendant drops at adsorption–desorption equilibrium during the volume reduction and expansion processes. Interfacial jamming and wrinkles were visible on the drop surface of Sample 1. (f) Schematic illustration of the self-assembled binary microgels at the interface in the presence of 0.5 vol% octanol. Sample 1 : 0.1 wt% PDEAEMA microgels (water) – 0.1 wt% PNIPAM-*co*-MAA microgels (oil) + 0.5 vol% octanol (oil); Sample 2 : 0.1 wt% PDEAEMA microgels (water) – 0.5 vol% octanol (oil); Sample 4: deionized water – 0.5 vol% octanol (oil); Sample 5: deionized water – toluene; Sample 6: 0.1 wt% PNIPAM-*co*-MAA microgels (water) – toluene; Sample 8 : 0.05 wt% PDEAEMA microgels (water) – 0.1 wt% PNIPAM-co-MAA microgels (oil) + 0.5 vol% octanol (oil); Sample 9 : 0.1 wt% PDEAEMA microgels (water) – 0.05 wt% PNIPAM-co-MAA microgels (oil) + 0.5 vol% octanol (oil).

The ζ potentials of PNIPAM-*co*-MAA microgels and PDEAEMA microgels demonstrate that these two types of microgel carried opposite charges across a wide range of pH values (pH 3–8.5), implying that there should be strong electrostatic attraction between them under these conditions (Fig. 4a). The absolute value of the ζ potential reduced when mixing oppositely charged particle dispersions together, due to charge neutralization (Fig. S13†). Indeed, after we dispersed these two types of microgels in the same aqueous medium by gentle shaking, they quickly aggregated to form a macro-condensate at the bottom of the vial (Fig. S14 and Movie S4†). The effect of pH values on Pickering emulsions stabilized by binary microgels was further investigated. Different from particle bilayer structures formed under neutral conditions, although stable inverse W/O Pickering emulsions can be prepared at a low pH value (pH 1), like-charged PDEAEMA microgels in this case preferred to disperse within the water droplet rather than attach to the interface, due to the presence of electrostatic repulsion. Thus, only a monolayer consisting of PNIPAM-*co*-MAA microgels was observed at the interface (Fig. S15a†). In contrast, due to the deprotonation of PNIPAM-*co*-MAA microgels with increasing hydrophilicity under alkaline conditions (pH 11), O/W Pickering emulsions with a partial W/O/W double emulsion morphology were formed in this case (Fig. S15b†).

### Interfacial properties of binary microgels

After incorporating oppositely charged PDEAEMA microgels into the water phase, the initial adsorption of microgels was further enhanced, and the interfacial tension rapidly reached equilibrium within approximately 20 seconds (Fig. 4b) due to the electrostatic driving force. PDEAEMA microgels thus integrated with PNIPAM-*co*-MAA microgels at the interface to form a bilayer structure (Fig. 3d). Fig. 4c shows the interfacial dilatational moduli of adsorption layers assembled from these microgels. As can be seen, sole PNIPAM-*co*-MAA or PDEAEMA microgels (Sample 2 and Sample 7, respectively) dispersed in the water phase could only form interfacial films with relatively low moduli, probably due to low surface coverage and weak in-plane interparticle interactions. Even though the strength of the microgel monolayer at the interface of Sample 3 was greatly increased when PNIPAM-*co*-MAA microgels were pre-swollen by octanol, the bilayer structure assembled from binary microgels (Sample 1) endowed the interfacial film with approximately twice the viscoelasticity (|E*| > 50 mN m^−1^) of the microgel monolayer (Sample 3).

The appearance of pendant drops during volume reduction and expansion is shown in Fig. 4e–f. The results indicate that sole PDEAEMA microgels could not form a strong interfacial film with a remarkable elastic response (Sample 2, Movie S5†), whereas wrinkles were clearly visible on the drop surface laden with binary microgels when only a small compressive force was applied to the interface (Sample 1, Movie S6†). Compared with the interface solely covered by octanol-swollen PNIPAM-*co*-MAA microgels, the binary microgel-coated interface showed a stronger elastic response under both compression and extension, which was mainly attributed to the formation of a denser and more rigid interfacial bilayer structure *via* electrostatic attraction. To the best of our knowledge, although similar wrinkles have been observed in insoluble monolayers from solid particles,^49^ lipids,^50,51^ and some specific proteins,^52,53^ such surprising and pronounced surface wrinkles are unprecedented at the interfaces laden with self-assembled soft microgels. This demonstrates the feasibility of using binary microgel-driven interfacial assembly as a rapid and highly controllable strategy for the construction of microgelsomes containing a non-covalent elastic interfacial layer.

In addition, it was found that changing the concentration of one microgel component in the biphasic system would alter the dynamic interfacial tension and generate an interface with different viscoelasticity (Fig. 4d). Generally, although the viscoelasticity of the interfacial layer depended on the concentration of both PNIPAM-*co*-MAA and PDEAEMA microgels, the presence of PNIPAM-*co*-MAA microgels played a main role in governing the interfacial structure as well as the rheology. Specifically, low concentrations of PNIPAM-*co*-MAA microgels were not able to fully cover the drop surface in a short time. In this case, the relatively higher concentration of PDEMEMA microgels led to their occupying a greater surface area, such that less vacant space was available for PNIPAM-*co*-MAA microgel adsorption. As a result, a well-defined interfacial bilayer structure could not be developed, which resulted in the poor elasticity of the drop surface containing low coverage of PNIPAM-*co*-MAA microgels.

### Electrostatic repulsion between like-charged particles

**Fig. 5:**
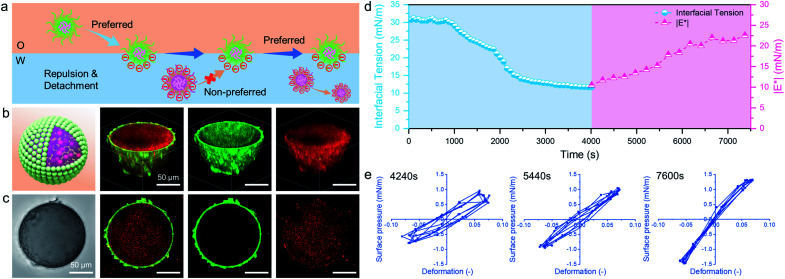
(a) Proposed stabilization mechanism of like-charged microgels and PS latex particles dispersed in different phases. (b) Schematic 3D model, 3D CLSM images and (c) corresponding optical microscopy image and the cross-sectional profile of a W/O emulsion droplet initially stabilized by 0.5 wt% PNIPAM-*co*-MAA microgels (green) in the oil phase, containing like-charged 0.5 wt% PS-*co*-MAA latex particles (red) in the water phase. (d) Dynamic interfacial tension and |E*| of the water–oil interface (0.5 vol% octanol + 99.5 vol% toluene) containing 0.1 wt% PNIPAM-*co*-MAA microgels in oil and 0.1 wt% PS-*co*-MAA latex particles in water. (e) Lissajous plots of surface pressure *versus* deformation for the water–oil interface stabilized by 0.5 wt% PNIPAM-*co*-MAA microgels in the presence of 0.5 wt% PS-*co*-MAA latex particles as a function of time, where A (surface area) = 20 mm^2^, dA/A (deformation amplitude) = 0.07 and *ω* (frequency) = 0.05 Hz.

In addition to the electrostatic attraction between oppositely charged microgels, we also evaluated and illustrated the interaction between like-charged particles. For this, we dispersed PNIPAM-*co*-MAA microgels in the two immiscible phases, respectively, or added poly(styrene-co-methacrylic acid) (PS-*co*-MAA) latex particles into the water phase with PNIPAM-*co*-MAA microgels in the oil phase. The results in Fig. 5b, c and S16† demonstrate that PNIPAM-*co*-MAA microgels in the oil phase were able to adsorb at the interface and form inverse W/O Pickering emulsions, whereas most like-charged microgels or PS particles were only able to disperse within water droplets. This mechanism is illustrated in Fig. 5a. First, the *in situ* modified microgels would occupy the interface because of their higher interfacial activity. In contrast, owing to the strong electrostatic repulsion between like-charged particles and the low interfacial activity of particles in the water phase, it is difficult for these particles encapsulated inside to integrate with the microgels with similar charges at the interface. The dynamic interfacial tension and elasticity of the system containing PNIPAM-*co*-MAA microgels and PS-*co*-MAA particles also demonstrate that the adsorption process of PNIPAM-*co*-MAA microgels is highly inhibited (Fig. 5d). It took longer to reach the interfacial tension equilibrium. Indeed, the presence of like-charged particles in the water phase would cause repulsion and hindered diffusion of microgels from the oil phase to the interface. The timescale was significantly prolonged, especially the initial diffusion portion of this process. A similar trend was seen in the elasticity of the drop surface, which underwent a very slow increase over a long measurement period (>2 h). The Lissajous plots depicted in Fig. 5e also illustrate the gradual formation of the microgel monolayer at the interface, with the elasticity of the layer increasing over the prolonged measurement time. It is worth noting that an external input of energy from emulsification can accelerate this adsorption process, which results in the dispersion of like-charged particles within water droplets.

### Selective protection and programmed release of bioactive substances

The encapsulation and release behaviours of colloidosomes rely primarily on their shell thickness, linkages, and the vacant space between neighbouring particles on the shell.^20^ Up to now, even though colloidosomes have been used as cargo storage and delivery systems, the precise and controllable release of multiple components from colloidosomes remained highly challenging.^54–56^ In addition, the rupture of the colloidosome structure is always required for the release of encapsulated components, which means that such processes are irreversible. However, as colloidosomes can be formed by combining two diverse microgels with complementary properties and the interfacial structures can be easily tailored, we envision that the fabricated microgelsomes were functionalized with space-time responsiveness that both long-term storage and rapid programmed release of multiple components can be achieved relying on the size-selective permeability.

**Fig. 6:**
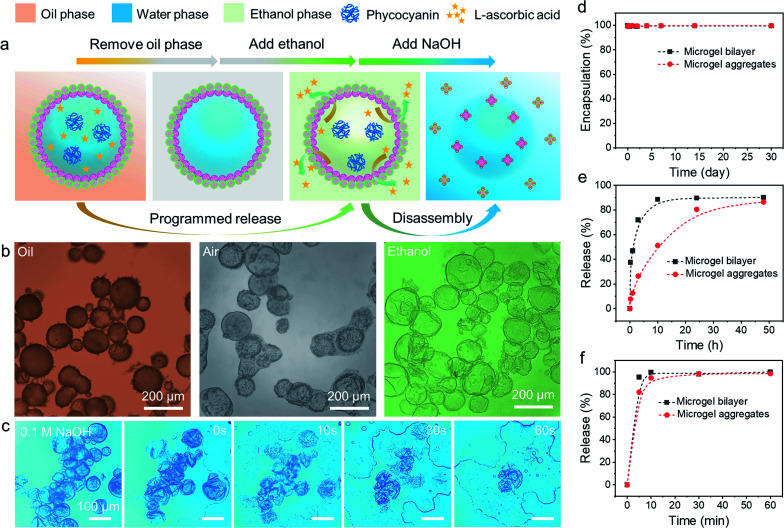
(a) Schematic illustration of the ethanol induced programmed release and alkali induced disassembly of binary microgel stabilized microgelsomes. (b) Optical microscopy images of the appearance of microgelsomes co-stabilized by PNIPAM-*co*-MAA microgels and PDEAEMA microgels during the oil removal and ethanol penetration processes. (c) Disassembly of microgelsomes in 0.1 M NaOH. (d) Encapsulation profile of phycocyanin by microgelsomes as a function of time. Programmed release profile of l-ascorbic acid (vitamin C) from microgelsomes (e) in water solution and (f) triggered by additional ethanol.

The stability and morphology of microgelsomes with an interfacial bilayer structure were further investigated in different mediums. As shown in Fig. 6b, after the removal of the oil phase, the elastic shell consisting of binary microgels could effectively support the capsules' structures and prevent their rupture. Ethanol was normally used as a demulsifier to induce droplet coalescence and the phase separation of emulsions, because it is miscible in both phases and can significantly reduce interfacial tension.^57^ In this work, we found ethanol that has a similar effect on the de-stabilization of sole PNIPAM-*co*-MAA microgel stabilized emulsions. Thus, O/W or W/O Pickering emulsions stabilized by PNIPAM-*co*-MAA microgels alone were rapidly broken after the introduction of ethanol (Fig. S17†). Interestingly, after binary microgels had combined at the interface *via* electrostatic interactions to form a compact physical barrier, the microgelsomes were highly stable in both air and ethanol. Therefore, we propose that the unique interfacial bilayer structure endows microgelsomes with the long-term storage and controlled release of various compounds. To evaluate the encapsulation and release behaviours of the as-prepared microgelsomes, phycocyanin (a protein) and l-ascorbic acid (vitamin C) were selected as model bioactive substances, due to their different sizes and intrinsic properties. As shown in Fig. 6d–f, phycocyanin as a macro-molecular protein can be well protected by encapsulation within the water core of the microgelsomes for over 1 month. This was attributed to the excellent stability and densely packed binary microgels shell of microgelsomes. In another case, vitamin C was first dissolved in the water phase before emulsification, as it is a small hydrophilic molecule. It was found that microgelsomes with a bilayer structure at the interface can rapidly release vitamin C in the first 3 hours. After 10 hours, almost all vitamin C was released outside. Surprisingly, the addition of ethanol caused the complete release of encapsulated vitamin C in less than 10 min, without destroying the colloidosome structure, which means that the polar ethanol molecules can alter the permeability of the microgel shell.

Compared to PDEAEMA microgels, poly(*N*-isopropylacrylamide-*co*-4-vinylpyridine) (PNIPAM-*co*-4VP) microgels which are also negatively charged while having a much smaller particle size were chosen to incorporate with positively charged PNIPAM-*co*-MAA microgels to form microgelsomes under the same conditions (Fig. S18 and 19†). It should be noted that oppositely charged PNIPAM-*co*-4VP microgels are smaller and able to lock on the free space between adjacent PNIPAM-*co*-MAA microgels. The aggregated shell can provide microgelsomes with tighter connection between various microgels and form smaller interstitial pores than the microgel bilayer. Therefore, the microgelsomes consisting of an aggregated layer show gradual release of vitamin C in a relatively long period (∼48 h). Meanwhile the encapsulation profile of phycocyanin and ethanol-triggered release profile of vitamin C were quite similar between these two types of microgelsomes have different interfacial structures.

The process and mechanism of selective encapsulation and programmed release are elucidated in Fig. 6a. The size of the encapsulated compounds is a key consideration. Specifically, the pore size in the shell of microgelsomes depends on the interaction and the size of both microgels. Thus, despite the interfacial bilayer consisting of relatively large PNIPAM-*co*-MAA microgels (∼1.2 μm) and PDEAEMA microgels (∼1.5 μm) (Fig. S20†), the interstitial pores at the interface should be relatively small because of the heterocoagulation of adjacent oppositely charged microgels. In addition, as soft microgels exhibit a typical core–corona morphology, the extension of the external corona of adsorbed microgels at the oil–water interface results in the formation of an interconnected polymer network, which is rationalized as another reason to promise the encapsulation and protection of macro-molecules. On the other hand, the swelling ratio of microgels in ethanol was larger than that in both octanol and toluene. This means that microgels have higher affinity towards ethanol and exhibit a larger swelling ratio when adding ethanol into the oil phase (Fig. S21†). Therefore, it is reasonable that microgelsomes in ethanol may be larger than that in oil and air. In addition, compared to octanol-swollen microgels, we suppose that ethanol-swollen microgels have larger internal channels for small molecule diffusion and the packing density of microgels was also reduce due to the expanded surface area. Hence, vitamin-C can be released much faster from the water core of microgelsomes when additional ethanol was introduced into the biphasic system. Since the bilayer shell of microgelsomes was constructed by the reversible physical crosslinking (electrostatic attraction) between microgels, the presence of ions in the dispersion medium may affect the interparticle interaction between microgels. We thus investigated the pH and salt responses of microgelsomes to NaOH, HCl and NaCl solutions of various concentrations. As shown in Fig. 6c and S22,† the disassembly of the microgels and the rupture of microgelsomes only occurred at a high pH value due to the strong electrostatic repulsion between adjacent microgels. In contrast, the microgelsomes retained well-defined shapes at a low pH value or high salt concentration. This can be accounted by the charge screening effect that hydrogen bonding and hydrophobic interactions were strengthened between PNIPAM-*co*-MAA microgels under acidic or high-salt conditions, which resulted in the intact microgel layer of microgelsomes.

We also encapsulated other bioactive substances, such as starch nanocrystals and enzymes, in PNIPAM-*co*-MAA microgels stabilized inverse W/O emulsions, to explore their interactions (Fig. S23†). It was found that depending on their surface charges and functional groups, bioactive substances were either selectively confined at the interface or rigorously protected inside emulsion droplets. Generally, if microgels and bioactive substances were like-charged, the bioactive substances can be well encapsulated inside due to electrostatic repulsion. Otherwise, they would be integrated with the microgels at the interface due to electrostatic attraction between complementary functional groups. This versatile strategy may have unique applications in the encapsulation of charged substances and in interfacial catalysis using certain enzymes.

## Conclusion

In summary, we have developed an original strategy to prepare inverse W/O Pickering emulsions using octanol-swollen microgels. *In situ* modified PNIPAM-*co*-MAA microgels exhibited distinctive interfacial behaviours and could achieve high surface coverage *via* a spontaneous self-assembly process. In addition, non-covalent microgelsomes with a unique interfacial bilayer structure can be formed by cooperating binary oppositely charged microgels at the interface. Based on the reconfigurable shell structure with selective permeability, large substances can be effectively encapsulated and protected for a long time, whereas the release of small molecules can be precisely controlled by using additional ethanol or microgels with different sizes to mediate the shell permeability. In addition, the use of suitable microgels enabled charged bioactive substances to be selectively protected within emulsion droplets or anchored at the interface, depending on the nature of the electrostatic interactions. This novel strategy of forming inverse emulsions stabilized by octanol-swollen microgels and templated microgelsomes overcomes the limitations of conventional microgel-stabilized O/W emulsions and offer new prospects for colloidosome research. In particular, it may enable the rational design and construction of novel non-covalent colloidosomes with a reconfigurable, semipermeable, stimulus-responsive and mechanically elastic microgel layer. In addition to applications preliminarily demonstrated in this work (*e.g.*, microencapsulation and programmed release), we envisage that such microgelsomes have the potential to be a useful protocell model system for enzyme catalysis and *in situ* protein synthesis.

## Data availability

All experimental supporting data and simulation details are available in the ESI.†

## Author contributions

X. G.: conceptualization, methodology, investigation, formal analysis, and writing – original draft. Y. L.: simulation and analysis. Z. L. W.: formal analysis, review & editing, and supervision. Y-L. S. T.: formal analysis, review & editing, and supervision. T. N.: conceptualization, writing – review & editing, project administration, and supervision.

## Conflicts of interest

There are no conflicts to declare.

## Supplementary Material

SC-013-D2SC01082H-s001

SC-013-D2SC01082H-s002

SC-013-D2SC01082H-s003

SC-013-D2SC01082H-s004

SC-013-D2SC01082H-s005

SC-013-D2SC01082H-s006

SC-013-D2SC01082H-s007
