# Supplementary material for: Non-covalent reconfigurable microgel colloidosomes with a well-defined bilayer shell
Source: Chem Sci. 2022 Apr 26;13(21):6205–16. doi: 10.1039/d2sc01082h (PMC9159095; doi:10.1039/d2sc01082h)
Supplement: SC-013-D2SC01082H-s007 [file SC-013-D2SC01082H-s007.pdf]

Electronic Supplementary Material (ESI) for Chemical Science.
This journal is © The Royal Society of Chemistry 2022

Supporting Information

Non-Covalent Reconfigurable Microgel Colloidosomes with a Well-Defined Bilayer Shell

Xin Guan [a], Yang Liu [a], Zhili Wan [a,b*], Ying-Lung Steve Tse [a*], and To Ngai [a*]

[a] Department of Chemistry, The Chinese University of Hong Kong, Shatin, N.T., Hong Kong, China

[b] School of Food Science and Technology, South China University of Technology, Guangzhou 510640, China

Table of Content

Experimental Procedures 3

Materials. 3
Synthesis of fluorescent PNIPAM-*co*-MAA microgels. 3
Synthesis of fluorescent PDEAEMA microgels. 3
Synthesis of fluorescent PS-*co*-MAA latex particles. 3
The purification of synthetic fluorescent PNIPAM-*co*-MAA microgels and PS-*co*-MAA latex particles. 3
Preparation of conventional O/W Pickering emulsions. 3
Preparation of inverse W/O Pickering emulsions solely stabilized by PNIPAM-*co*-MAA microgels. 4
In situ preparation of microgelsomes co-stabilized by binary microgels based on electrostatic attraction. 4
Encapsulation and anchoring of nanoparticles, bio-based nanocrystals and enzyme in inverse W/O Pickering emulsion systems. 4
The effect of interfacial structure of microgelsomes on their encapsulation and controlled release performances. 4
Dynamic interfacial tension, interfacial rheology measurement and contact angle measurement. 4
Characterization. 5
Simulation details. 5

Results and Discussion 6

Table. S1 System composition of hydrogen bond systems. 6
Table. S2 System composition of oil-water interface systems. 6
Fig. S1 Change of emulsion type with different octanol concentrations. Pickering emulsions stabilized by 1 wt% PNIPAM-*co*-MAA microgels dispersed in (a) oil and (b) water, respectively. 7
Fig. S2 Photographs and confocal laser scanning microscope (CLSM) images of Pickering emulsions (W:O=1:1, v/v) stabilized by 1 wt% PNIPAM-*co*-MAA microgels in water phase initially containing (a) 0 vol% octanol, (b) 20 vol% octanol, (c) 50 wt% octanol and (d) 100 vol% in the oil phase. 7
Fig. S3 The residual octanol in the oil phase after the adsorption by different concentration of PNIPAM-*co*-MAA microgels. The initial concentration of octanol is 500 ppm. 8
Fig. S4 Three-phase contact angle of water drop for swollen PNIPAM-*co*-MAA microgel layer in the absence or presence of different octanol concentration. (a) toluene, (b) 1 vol% octanol, (c) 5 vol% octanol, and (d) 10 vol% octanol. 8
Fig. S5 (a) TEM images and (b) CLSM images of aggregated PNIPAM-*co*-MAA microgels dispersed in toluene. 9
Fig. S6 Dynamic interfacial tension of the water-oil interface containing different concentrations of octanol, which indicates the solvent effect of octanol in these biphasic systems. 9
Fig. S7 (a) Photographs of the appearance of PNIPAM-*co*-MAA microgels dispersion in octanol during long time storage. (b) The CLSM image and (c) size distribution of PNIPAM-*co*-MAA microgels dispersed in octanol. The size of microgels in octanol is around 800 nm which is slightly smaller than dispersed in water. 10
Fig. S8 The appearance of the pendant drop in octanol containing 0.1 wt% PNIPAM-*co*-MAA microgels at adsorption-desorption equilibrium during the volume reduction and expansion process. No interfacial jamming and wrinkles appeared on the drop surface. 10
Fig. S9 Lissajous plots of surface pressure versus deformation for a water-oil interface stabilized by (a) 0.1 wt% PNIPAM-*co*-MAA microgels in water, (b) 0.1 wt% PDEAEMA microgels in water, (c) 0.1 wt% PNIPAM-*co*-MAA microgels in oil containing 0.5 vol% octanol, and (d) the combination of 0.1 wt% PNIPAM-*co*-MAA microgels in oil containing 0.5 vol% octanol and 0.1 wt% PDEAEMA microgels in water. A = 20 mm^2, deformation amplitude 0.05, ω = 0.05 Hz. 11
Fig. S10 CLSM images of O/W Pickering emulsions stabilized by 1 wt% PDEAEMA microgels at pH 7 with oil-water ratio of (a) 1:1 and (b) 2:1. 12
Fig. S11 Water contact angle of PDEAEMA microgels coated silicon wafers at different pH values. 12

Fig. S12 CLSM images of W/O Mickering emulsions stabilized by 0.5 wt% PNIPAM-*co*-MAA microgels (red) in oil phase initially, containing 0.5 wt% PS-NH4+ latex particles (green). 12
Fig. S13 ζ potential measurements of different microgels, PS latex particles and the mixture of PNIPAM-*co*-MAA microgels (0.05 wt%) and PDEAEMA microgels (0.01 wt%) used in this work. 13
Fig. S14 The mixing process of PNIPAM-*co*-MAA microgels and PDEAEMA microgels with opposite charges. The sedimentation of particle aggregates indicates the exist of strong electrostatic attraction. 13
Fig. S15 CLSM images of Pickering emulsions stabilized by 0.5 wt% PNIPAM-*co*-MAA microgels (red) in the oil phase and 0.5 wt% PDEAEMA microgels (red) in the water phase at different pH values. (a) pH 1, (b) pH 11. 14
Fig. S16 CLSM images of W/O Pickering emulsions stabilized by 0.5 wt% PNIPAM-*co*-MAA microgels (green) in the oil phase, encapsulating 0.5 wt% PNIPAM-*co*-MAA microgels (red) in the water phase, respectively. 14
Fig. S17 Schematic illustration and optical microscopy images of (a) conventional O/W Pickering emulsion and (b) inverse W/O Pickering emulsion stabilized by 1 wt% PNIPAM-*co*-MAA microgels in water and oil phase respectively, and the demulsification of emulsions induced by additional ethanol. 15
Fig. S18 Microstructure of PNIPAM-*co*-4VP microgel, PNIPAM-*co*-MAA microgel and corresponding complex particle consists of larger PNIPAM-*co*-MAA microgels as the core and smaller PNIPAM-*co*-4VP microgels as the corona. 15
Fig. S19 Schematic illustration and morphology of microgelsomes with aggregated layer composed of oppositely charged PNIPAM-*co*-MAA microgels and PNIPAM-*co*-4VP microgels in toluene and ethanol. 16
Fig. S20 Microstructure and hydrodynamic diameter of varies microgels and PS latex particles. 16
Fig. S21 Mean particle size of PNIPAM-*co*-MAA microgels in different dispersion mediums. 16
Fig. S22 The pH and salt response of microgelsomes with particle bilayer consisted of PNIPAM-*co*-MAA microgels and PDEAEMA microgels in the solutions containing different concentrations of HCl and NaCl. 17
Fig. S23 W/O Pickering emulsions stabilized by negativly charged 0.5 wt% PNIPAM-*co*-MAA microgels (red) in the oil phase, containing (a) 0.5 wt% Calcofluor white stained starch nanocrystals (blue) with negative charges, and (b) 0.5 wt% FITC stained lipase (green) with positively charged residues (e.g., amino groups) on the surface in water solution. 17

References 17

Experimental Procedures

Materials.

N-Isopropylacrylamide (NIPAM, 99%), methacrylic acid (MAA, 99%) and fluorescein isothiocyanate (FITC, 90%) were bought from J&K Scientific. 2-(Diethylamino)ethyl methacrylate (DEAEMA), ethylene glycol dimethacrylate (EGDMA, >97%), N-(3-dimethylaminopropyl)-N′-ethylcarbodiimide hydrochloride (EDC, 98%), dodecane (99%) and phycocyanin from *Spirulina* were obtained from TCI Co., Ltd. N-hydroxysuccinimide was purchased from Aladdin. Fluoresceinamine, isomer I, divinylbenzen (DVB, 80%), lipase from *Candida* sp. expressed in *Aspergillus oryzae*, 1-octanol (99%), dichloromethane (DCM) and sodium hydroxide (≥98%) were from Sigma-Aldrich. *N, N′*-Methylenebisacrylamide (MBA, 99%), (vinylbenzyl)trimethylammonium chloride (VBTMAC, >97%), perylene (99%) were from Acros Organics. Styrene (99%) and potassium persulfate (KPS) and L-ascorbic acid (vitamin C, 99%) were from Dieckmann Chemical Industry Co., Ltd. 2,2′-Azobis(2-methylpropionamidine) dihydrochloride (AIBA, 95%) was received from FUJIFILM Wako Pure Chemicals LTD. Methacryloxyethyl thiocarbonyl rhodamine B (PolyFluor® 570) was received from Polysciences, Inc. Toluene (99.5%) was received from Duksan Reagents Co., Ltd. Triethylamine (99%) was obtained from Scharlau. Hydrochloric acid (37%) was obtained from VWR International. All the chemicals were directly used without any further purification. Deionized water (Milli-Q grade, 18.2 MΩ cm) was used in all experiments for this work. All aqueous solutions with different pH values were prepared with the deionized water.

Synthesis of fluorescent PNIPAM-*co*-MAA microgels.

Typically, 2.4 g monomer (NIPAM) were firstly dissolved into 30 mL deionized water and then transferred to a 250 mL three-neck bottle flask. Then, 0.064 g crosslinker (MBA), 600 μL co-monomer (MAA) and 1 mL PolyFluor® 570 solution [1 mg/mL, 10%(v/v) ethanol solution] were added into monomer solution followed by nitrogen gas bubbling for 30 mins. During the de-gas process, the temperature of water bath increased from room temperature to 60°C. After that, 2 mL initiator solution containing 0.11 g KPS dissolved in deionized water was injected into the reaction mixture to initiate polymerization. Subsequently, the reaction solution turned from transparent to translucent in few mins and then to totally opaque in 1 h. The complete synthesis of PNIPAM-*co*-MAA microgels lasted for about 5 h.

To endow green fluorescence to PNIPAM-*co*-MAA microgels. 10 mL non-stained microgels with COOH groups (0.5 wt%) were firstly activated by 15 mg NHS and 20 mg EDC for 20 min at pH 5. Then TEA was added into the reaction solution to adjust the pH value around 7.4 followed by adding 0.25 mg fluoresceinamine to react with COOH groups in microgels. The reaction would last for 12 h to obtain the green fluorescent PNIPAM-*co*-MAA microgels

Synthesis of fluorescent PDEAEMA microgels.

Specifically, 0.5 g VBTMAC was firstly dissolved into 100 mL deionized water in a 250 mL three-neck round flask. Then, 10 mL DEAEMA and 100 μL EGDMA were introduced in the flask with 1mL PolyFluor® 570 solution [1 mg/mL, 10%(v/v) ethanol solution]. The monomer solution was de-gassed by bubbling nitrogen gas for 30 mins. After increasing water bath temperature up to 60°C, 3 mL initiator solution containing 100 mg AIBA was added in the reactant system to initiate polymerization. The polymerization reaction was carried on for 24 h under magnetic stirring (450 rpm) before the collection and purification of products.

Synthesis of fluorescent PS-*co*-MAA latex particles.

For the synthesis of PS latex particles with COOH functional groups, 5.5 mL styrene, 0.1 g DVB, 250 μL MAA and 5mL PolyFluor® 570 solution [1 mg/mL, 10%(v/v) ethanol solution were dissolved in 140 mL deionized water and then transferred into a 250 mL two-necked round flask with nitrogen gas bubbling inlet and outlet for 1 h. The reactant solution kept stirring at 400 rpm by using a magnetic stir bar. After increasing the temperature of water bath from room temperature to 70°C, 10 mL initiator solution containing 0.15 g KPS was injected into the reaction medium to initiate the reaction. The reaction was conducted in 70°C for 24 h to finish the polymerization.

The purification of synthetic fluorescent PNIPAM-*co*-MAA microgels and PS-*co*-MAA latex particles.

All synthesized microgels and PS latex particles were collected and purified by high spped centrifugation to remove the unreacted monomers, oligomers, unreacted initiator and fluorescent dyes. Specifically, PNIPAM-*co*-MAA microgels were purified by centrifucation at 10000 rpm for 20 min. The centrifugation and re-dispersion would conducted for at least five times to get the purified PNIPAM-*co*-MAA microgel dispersion. Finally, the purified microgel dispersion was freeze-drying to get the lyophilized microgels for following experiments. Similarly, the dispersion of PS-*co*-MAA latex particles was centrifuged at 12000 rpm for 10 min. The whole centrifugation and re-dispersion process would repeat for four times to get the particle dispersion.

Preparation of conventional O/W Pickering emulsions.

For the preparation of conventional O/W Pickering emulsions. The concentration of PNIPAM-*co*-MAA microgels or PDEAEMA microgels in water phase was adjusted to 1 wt% before use. After that, same volume of oil phase (e.g., toluene and dodecane) was added into the emulsification system. Then the two immisable liquids were mixed together by vortexing at 2700 rpm for 1 min to form conventional O/W Pickering emulsions.

Preparation of inverse W/O Pickering emulsions solely stabilized by PNIPAM-*co*-MAA microgels.

The preparation of inverse W/O Pickering emulsions involved the special treatment of PNIPAM-*co*-MAA microgels. In detail, PNIPAM-*co*-MAA microgels were firstly freeze-drying under vacume to prepare lyopilized microgels. The *in situ* modification of microgels can be achieved by two strategies. The first one is using a slight of octanol to swollen microgels powders before dispersing into an apolar oil phase such as dodecane and toluene. Another strategy is directly adding microgel powders into apolar solvents followed by adding a low concentration of octanol. Both of the two methods can facilitate the hydrophobilized modification of PNIPAM-*co*-MAA microgels. Typically, PNIPAM-*co*-MAA microgels (1 wt%) and octanol (10 vol%) were added into the oil phase followed by ultrasonification till microgels were well modified and dispersed in oil phase. Then, same volume of deionized water was added and mixed with the oil phase which was stained by perylene. The final W/O Pickering emulsion was obtained by vortexing at 2700 rpm for 1 min.

***In situ* preparation of microgelsomes co-stabilized by binary microgels based on electrostatic attraction.**

The fabrication of microgelsomes was based on the preparation of inverse W/O Pickering emulsions (W:O = 1:1, v/v) by voetxing at 2700 rpm. Binary microgels were introduced in a special Pickering emulsion system which was utilized as the template for further microgelsomes fabrication. Typically, the oil phase contained 0.5 wt% PNIPAM-*co*-MAA microgels with 10 vol% octanol, and the water phase contained 0.5 wt% PDEAEMA microgels with opposite charges. After emulsifying these two immiscible liquids by vortexing, two type of microgels with opposite charges would like to self-assemble onto the interface thus constructing ordered interfacial bilayer structure based on electrostatic attraction. The strong connection between microgels can help fabricate microgelsomes based on reversable physical bonding.

Encapsulation and anchoring of nanoparticles, bio-based nanocrystals and enzyme in inverse W/O Pickering emulsion systems.

Different kinds of substances including PNIPAM-*co*-MAA microgels and PS latex particles, starch nanocrystals and lipase were used to investigate the encapsulation and anchoring effect of inverse W/O Pickering emulsions. Generally, 0.5 wt% negatively charged PNIPAM-*co*-MAA microgels were dispersed in the perylene stained oil phase initially and another substance was added into the water phase before emulsification. After emulsification by vorteing at 2700 rpm at neutral condition, like-charged substance would encapsulated inside emulsion droplets because of electrostatic repulsion. On the other hand, oppositely charged substance would anchor on the interface because of electrostatic attraction.

The effect of interfacial structure of microgelsomes on their encapsulation and controlled release performances.

The encapsulation and controlled release performances of microgelsomes were conducted by using phycocyanin and vitamin C as model components. Microgelsomes with particle bilayer were prepared by using 0.5 wt% PNIPAM-*co*-MAA microgels in oil phase and 0.5 wt% PDEAEMA in water phase. 1 wt% phycocyanin and 0.045 wt% vitamin C were dispersed or dissolved in water phase initially. The emulsification involved mixing water phase with oil phase together (oil:water = 3:1, v/v) by vortexing at 2700 rpm for 1 min. Then, the corresponding microgelsomes floated on the surface of oil phase (DCM), followed by the careful addition of 4 mL of deionized water. To aviod the oxidation, the pH value of deionized water was adjusted to 3 for the release detection of vitamin C. For ethanol induced programmed release of vitamin C, 4 mL ethanol was used to substitude water as the receptor phase. The glass vials containing microgelsomes were kept at 25 °C in a shaking chamber, and 100 μL of the supernatants was periodically extracted for the subsequent UV−visible measurement.

Dynamic interfacial tension, interfacial rheology measurement and contact angle measurement.

The dynamic interfacial tension (IFT) and interfacial rheology between oil and water as well as water contact angle of different microgels were measured by an all-purpose contact angle measuring & contour analysis system (OCA 25, Dataphysics) at room temperature (25 °C) and analyzed with SCA 20 software. Briefly, "pendent drop" method was applied to measure the IFT and interfacial rheology. For example, a 10 μL of water drop was injected at 15 μL/s from the syringe and suspended in the oil containing 0.1 wt% PNIPAM-*co*-MAA microgels and 0.5 vol% octanol for continuous measurement. The shape of droplet was captured and analyzed by Young-Laplace equation to get the dynamic IFT. Based on the recorded instantaneous IFT and surface area, the interfacial dilational rheology of microgel self-assembled oil–water interface was measured by calculating dilational moduli. The interface was measured at surface area (A) of 20 mm^2, deformation amplitude (dA/A) of 0.07 or 0.05, frequency (ω) of 0.05 Hz for at least 2 h. The dilational modulus |E*| was determined through the equation below:

$$|E*| = A(d\gamma/dA)$$

Where A represents the surface area of the droplet, dA and dγ are the surface area change and interfacial tension change during the measurement, respectively. The interface was measured within the viscoelastic range with neglectable inertia under the measurement parameters.

"Sessile drop" method was used to obtain the water contact angle between pure water and microgel surface in both air and oil. Before the contact angle measurement, the concentration of PNIPAM-*co*-MAA microgels and PDEAEMA microgels in dispersions was diluted to 1 wt%. Then microgel dispersions was dripped on the surface of silicon wafer and dried at room temperature for 4 h to evaporate water. After dripping and drying,

a multi-layer of microgels was deposited on the substrates. For water contact angle in the air, a 5 μL water drop at different pH values was dripped and stayed on the wafer surface in the air. To obtain the three-phase contact angle of microgels, the silicon wafer coated with microgel multilayers were soaked in the oil solution containing different concentration of octanol before measurement. Afterwards, 5 μL water drop was dripped on the wafer surface in the oil. The shape of water drop was captured, and the water contact angle can be automatically calculated by SCA 20 software. All contact angle measurement was repeated 3 times on the same substrates. The error bar comes from the difference between the left and right contact angle by computer calculation.

Characterization.

The optical microscopy images of the prepared emulsions were obtained with an optical microscope (OLYMPUS, Japan). The confocal micrographs were taken with a Nikon Eclipse Ti inverted microscope (Nikon, Japan). The confocal laser scanning microscope (CLSM) was equipped with a 40×oil immersion objective. Perylene, FITC, and rhodamine B were excited by a laser with wavelength 408, 488 and 543 nm, respectively. The emulsions were placed on the cover glasses and a series of x/y layers was scanned. Dynamic light scattering (DLS) and ζ potentials were done by Zetasizer Nano ZS90 (Malvern). For SEM observation, microgels and particles were fully dried and then sputter-coated with a thin layer of Au prior to imaging using a Quanta 400F (FEI Company) scanning electron microscope equipped with a field emission electron gun at 10 kV. The concentrations of octanol as a function of microgels amount were determined using GC-2010 Plus (Shimadzu, Japan) equipped with a flame ionization detector (FID) on a column (AT-WAX column, 30.0 m length, 0.53 mm inner diameter, 1.00 μm film thickness). The encapsulation and release experiments were measured by using a UV−visible spectrometer (Alpha-1, Shanghai Lab-spectrum Instruments Co., Ltd.) to calculate the absorbance at 615 nm and 260 nm, respectively.

Simulation details.

We carried out all the simulations using the GPU-accelerated OpenMM (7.5.0) simulation package[1, 2] and the CHARMM Generalized Force Field (CGenFF)[3]. Langevin Integrator was applied and the temperature was maintained at 298 K. The time step was 2 fs. The Lennard-Jones interactions were smoothly switched off between 10 and 12 Å by a forced-based switching function. Long-range electrostatic interactions were calculated using the particle-mesh Ewald (PME) method[4] with an error tolerance of 10^(-5). The polymer parameters were generated by the CHARMM-GUI polymer builder[5] and the starting structure was built with the Packmol package[6].

For systems investigating hydrogen bonds, a polymer was solvated in the oil box (9nm×9nm×9nm) and the system compositions are shown in **Table S1**. The isotropic simulation cells were maintained by the Monte Carlo barostat at 1 bar. The first 10 ns simulation was discarded in the analysis process. For labelling the polymers, the two numbers in the parentheses represent the numbers of the two types of monomers in the diblock copolymer, respectively. If there is only one number in the parentheses, it is then the number of that only monomer type.

In the oil/water interface simulations, the size of the box was kept at 4.44nm×4.44nm×8.21nm and the interfaces located at around z = 0 nm and z = 3 nm. The system compositions are shown in **Table S2**. In each simulation, different numbers of octanol were added to the system, shown in **Fig. 2f$_3$**. The number of toluene molecules was adjusted to maintain a similar volume. During equilibration, the polymers were restrained at the center of the oil region in the z-axis (perpendicular to the interface) with a force constant of 1000 kJ/mol^{-1} under constant external pressure at 1 bar for 3 ns. In the production run, constant NVT dynamics was applied without a barostat, and the restraints on the polymers were removed. 30 simulations runs were carried out for each system and 3 polymers were included in each simulation. In **Fig. 2f$_3$**, we considered each polymer separately in a certain simulation, even though polymers may form aggregates and affect the dynamics on each other before reaching the interface. Therefore, each point in **Fig. 2f$_3$** was computed based on the 90 polymers in 30 runs (3 interacting polymers in each run).

Results and Discussion

Table. S1 System composition of hydrogen bond systems.

System	Toluene	Octanol	Polymer
PNIPAM-*co*-MAA	3177	10	1
PNIPAM	3438	10	1

Table. S2 System composition of oil-water interface systems.

System	Toluene surface coverage (%)	water	polymer	toluene	Octanol
PNIPAM-*co*-MAA (3-1)	0	1670	3	591	0
PNIPAM-*co*-MAA (3-1)	30	1670	3	579	8
PNIPAM-*co*-MAA (3-1)	52	1670	3	571	14
PNIPAM-*co*-MAA (3-1)	81	1670	3	560	22
PNIPAM-*co*-MAA (3-1)	100	1670	3	548	30
PNIPAM-*co*-MAA (6-2)	0	1670	3	591	0
PNIPAM-*co*-MAA (6-2)	22	1670	3	582	6
PNIPAM-*co*-MAA (6-2)	37	1670	3	576	10
PNIPAM-*co*-MAA (6-2)	60	1670	3	568	16

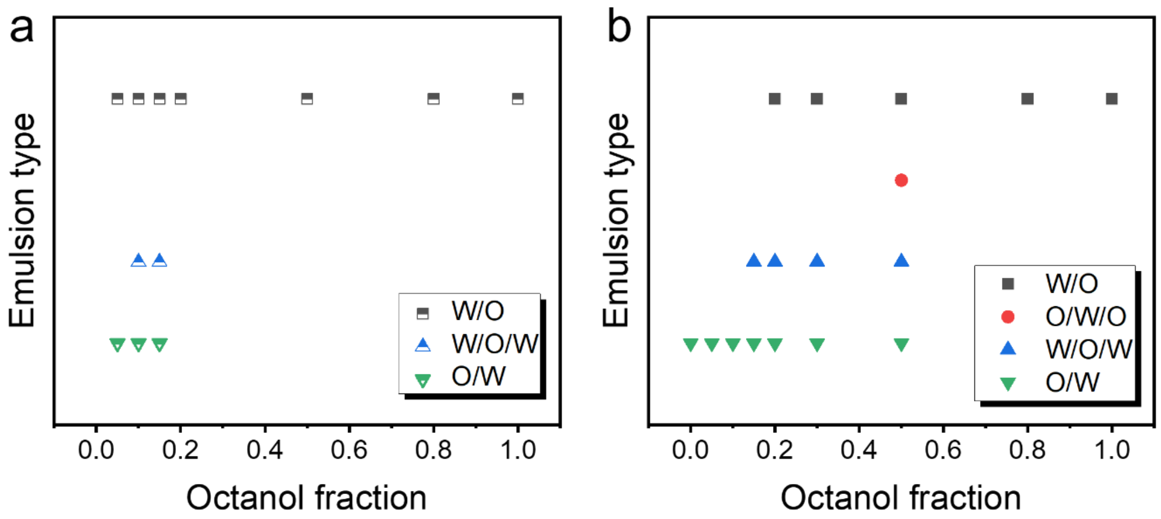

Fig. S1 Change of emulsion type with different octanol concentrations. Pickering emulsions stabilized by 1 wt% PNIPAM-*co*-MAA microgels dispersed in (a) oil and (b) water, respectively.

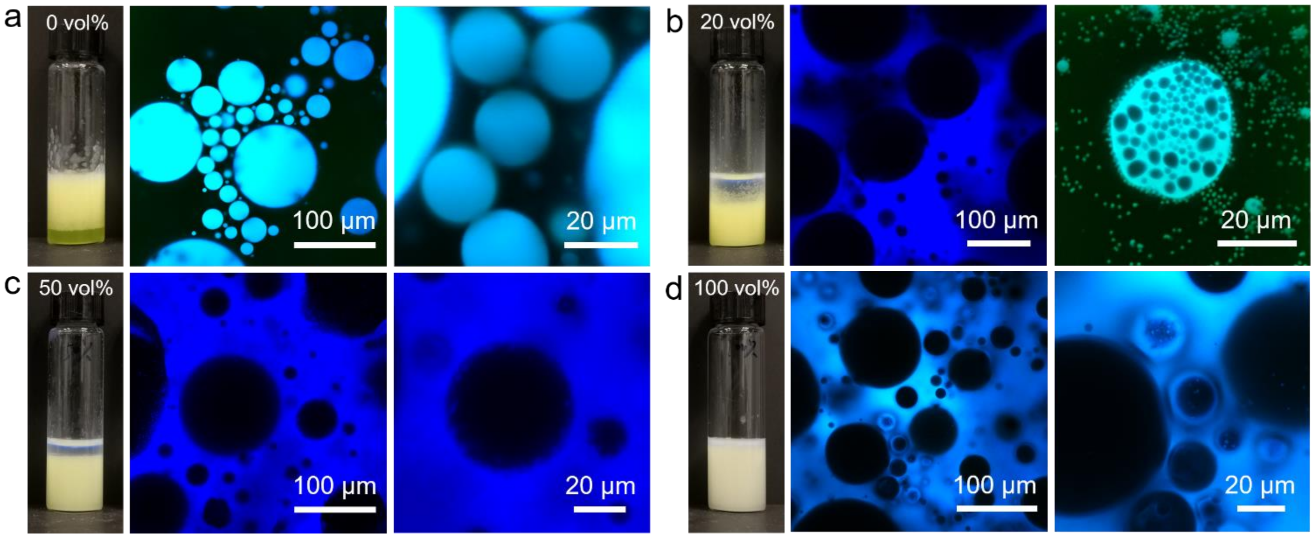

Fig. S2 Photographs and confocal laser scanning microscope (CLSM) images of Pickering emulsions (W:O=1:1, v/v) stabilized by 1 wt% PNIPAM-*co*-MAA microgels in water phase initially containing (a) 0 vol% octanol, (b) 20 vol% octanol, (c) 50 wt% octanol and (d) 100 vol% in the oil phase.

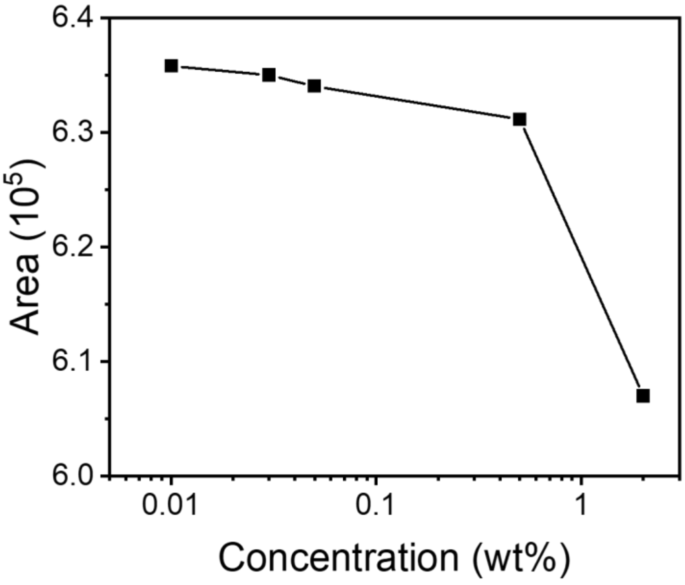

Fig. S3 The residual octanol in the oil phase after the adsorption by different concentration of PNIPAM-*co*-MAA microgels. The initial concentration of octanol is 500 ppm.

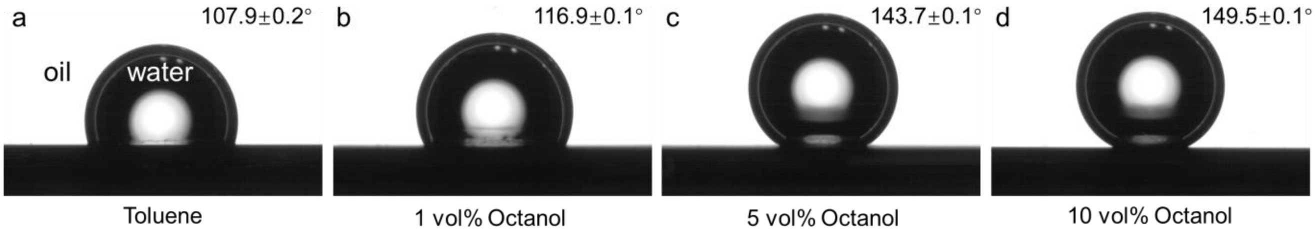

Fig. S4 Three-phase contact angle of water drop for swollen PNIPAM-*co*-MAA microgel layer in the absence or presence of different octanol concentration. (a) toluene, (b) 1 vol% octanol, (c) 5 vol% octanol, and (d) 10 vol% octanol.

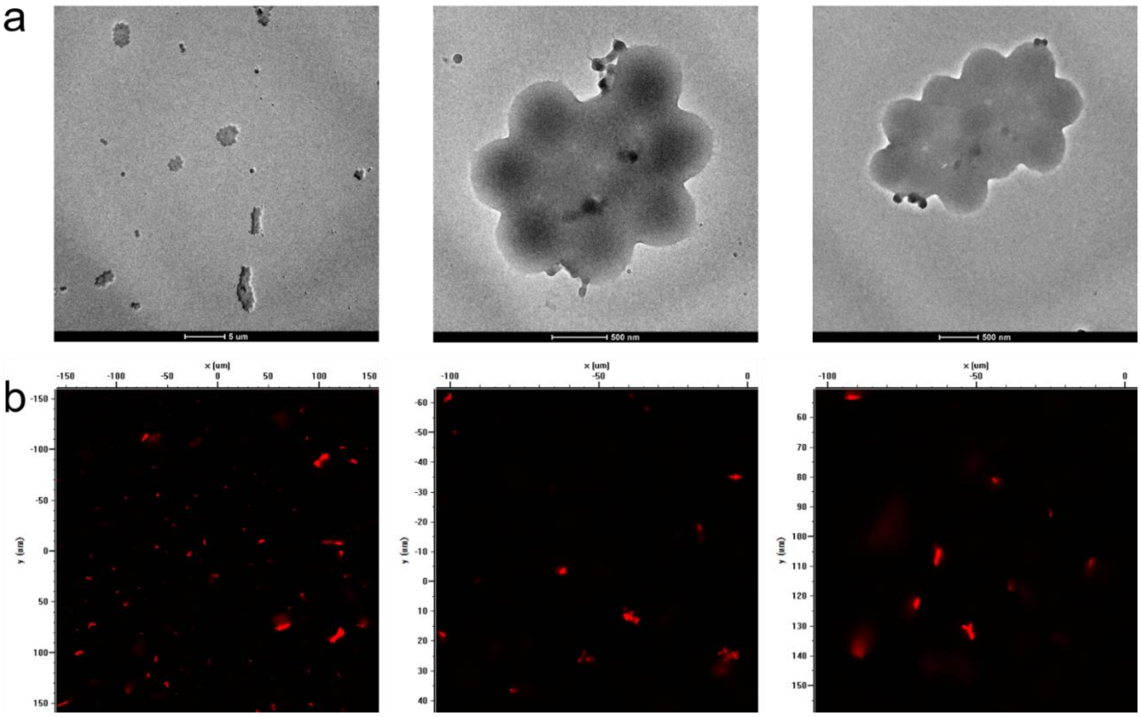

Fig. S5 (a) TEM images and (b) CLSM images of aggregated PNIPAM-*co*-MAA microgels dispersed in toluene.

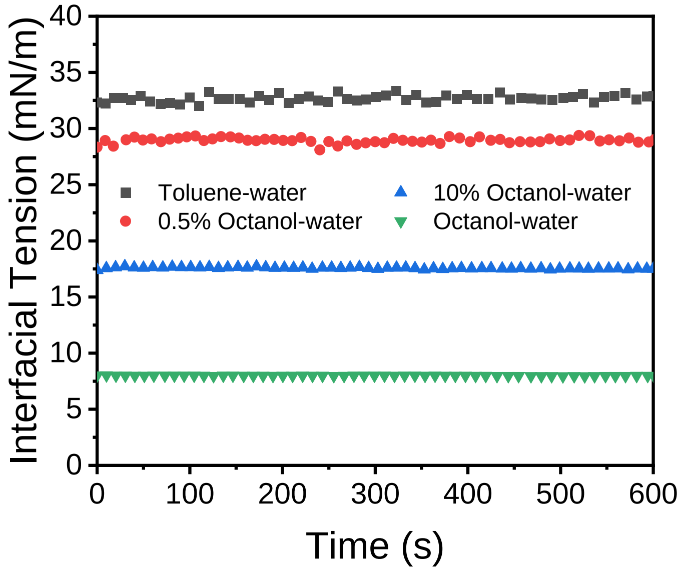

Fig. S6 Dynamic interfacial tension of the water-oil interface containing different concentrations of octanol, which indicates the solvent effect of octanol in these biphasic systems.

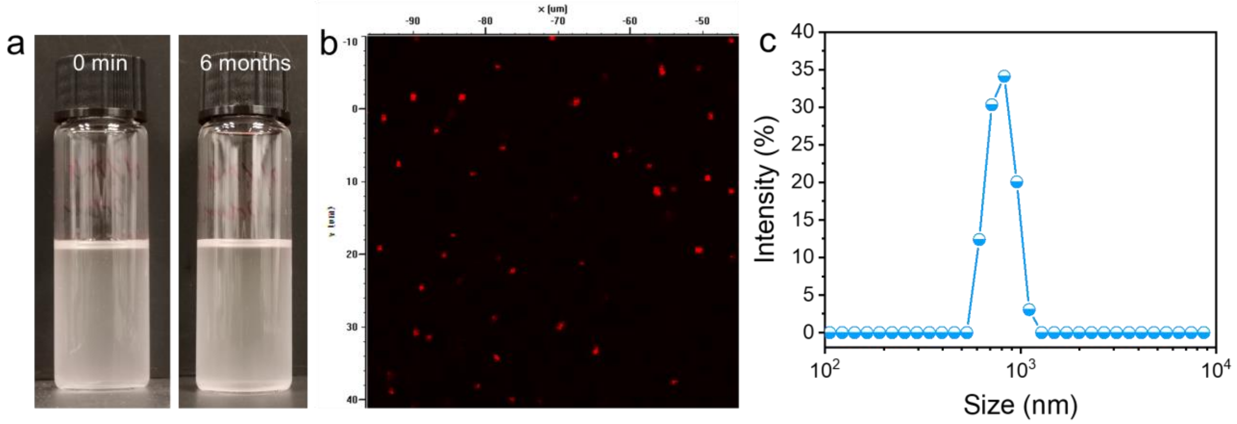

Fig. S7 (a) Photographs of the appearance of PNIPAM-*co*-MAA microgels dispersion in octanol during long time storage. (b) The CLSM image and (c) size distribution of PNIPAM-*co*-MAA microgels dispersed in octanol. The size of microgels in octanol is around 800 nm which is slightly smaller than dispersed in water.

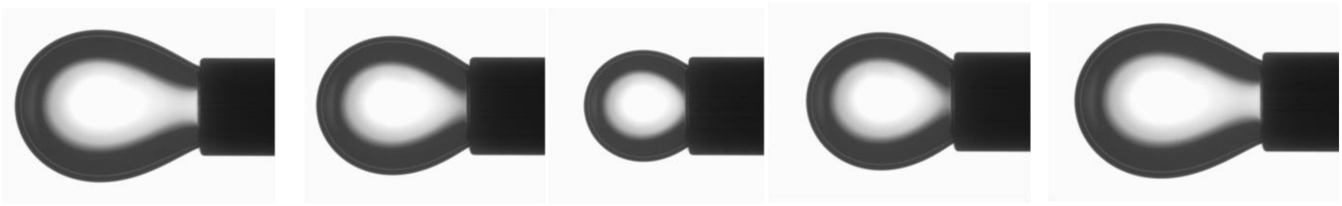

Fig. S8 The appearance of the pendant drop in octanol containing 0.1 wt% PNIPAM-*co*-MAA microgels at adsorption-desorption equilibrium during the volume reduction and expansion process. No interfacial jamming and wrinkles appeared on the drop surface.

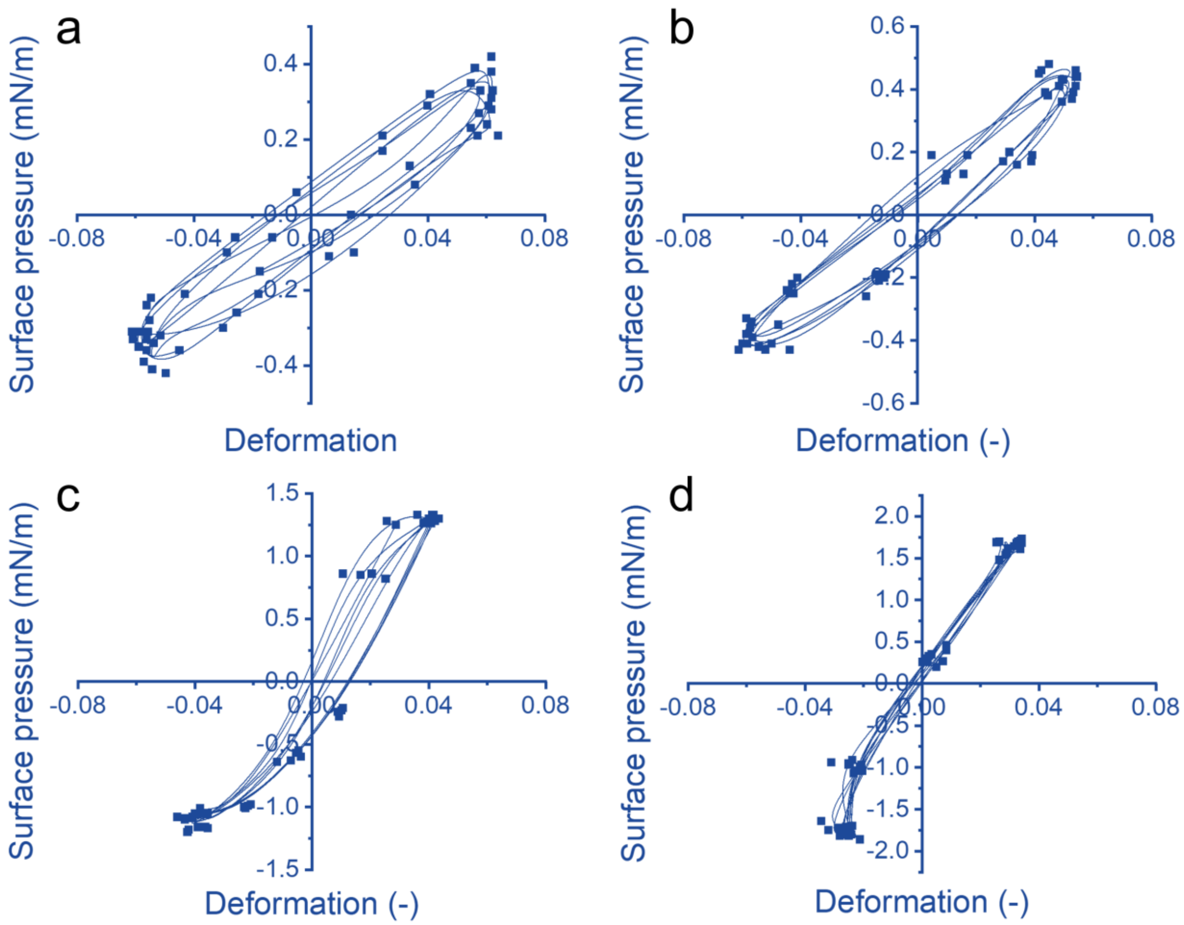

Fig. S9 Lissajous plots of surface pressure versus deformation for a water-oil interface stabilized by (a) 0.1 wt% PNIPAM-*co*-MAA microgels in water, (b) 0.1 wt% PDEAEMA microgels in water, (c) 0.1 wt% PNIPAM-*co*-MAA microgels in oil containing 0.5 vol% octanol, and (d) the combination of 0.1 wt% PNIPAM-*co*-MAA microgels in oil containing 0.5 vol% octanol and 0.1 wt% PDEAEMA microgels in water. A = 20 mm^2, deformation amplitude 0.05, ω = 0.05 Hz.

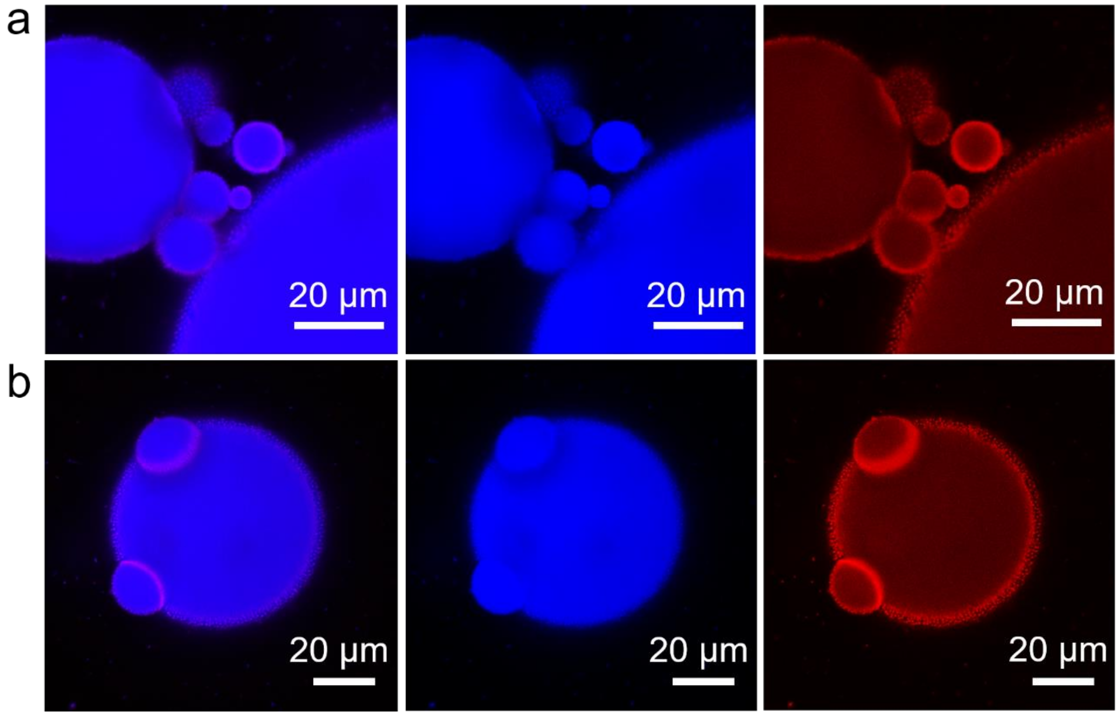

Fig. S10 CLSM images of O/W Pickering emulsions stabilized by 1 wt% PDEAEMA microgels at pH 7 with oil-water ratio of (a) 1:1 and (b) 2:1.

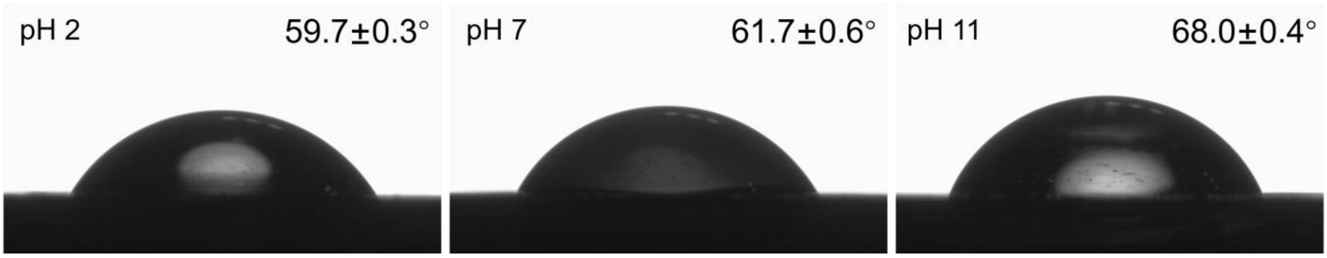

Fig. S11 Water contact angle of PDEAEMA microgels coated silicon wafers at different pH values.

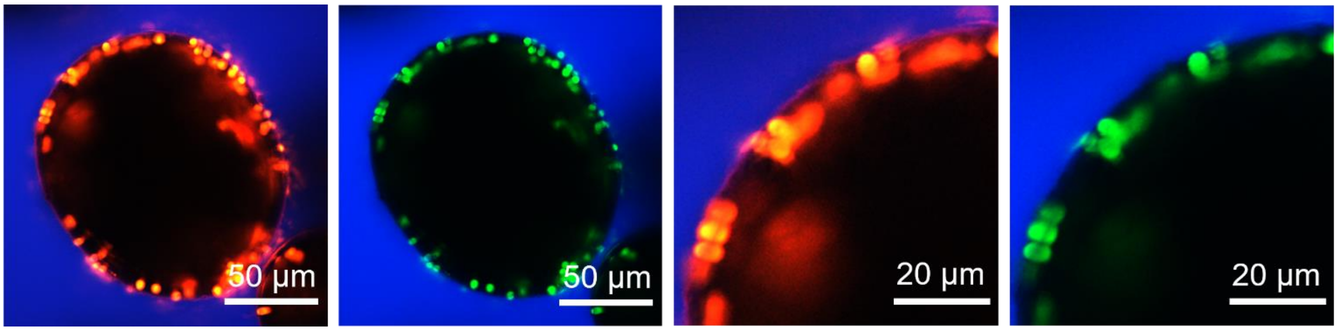

Fig. S12 CLSM images of W/O Mickering emulsions stabilized by 0.5 wt% PNIPAM-*co*-MAA microgels (red) in oil phase initially, containing 0.5 wt% PS-NH4+ latex particles (green).

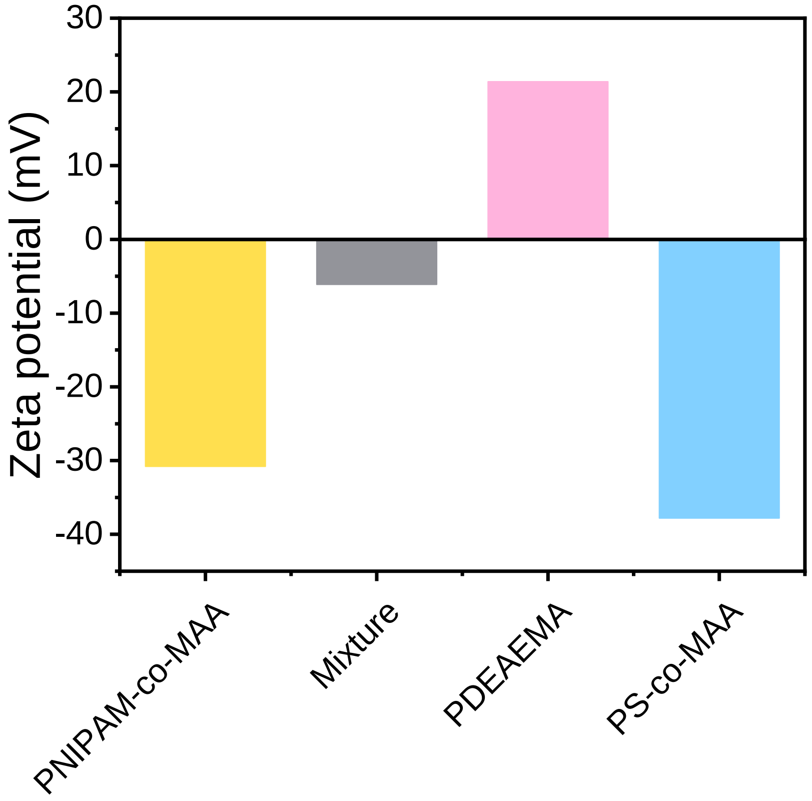

Fig. S13 ζ potential measurements of different microgels, PS latex particles and the mixture of PNIPAM-*co*-MAA microgels (0.05 wt%) and PDEAEMA microgels (0.01 wt%) used in this work.

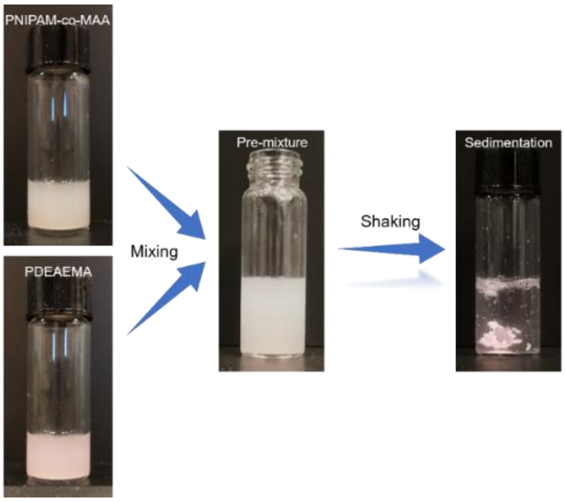

Fig. S14 The mixing process of PNIPAM-*co*-MAA microgels and PDEAEMA microgels with opposite charges. The sedimentation of particle aggregates indicates the exist of strong electrostatic attraction.

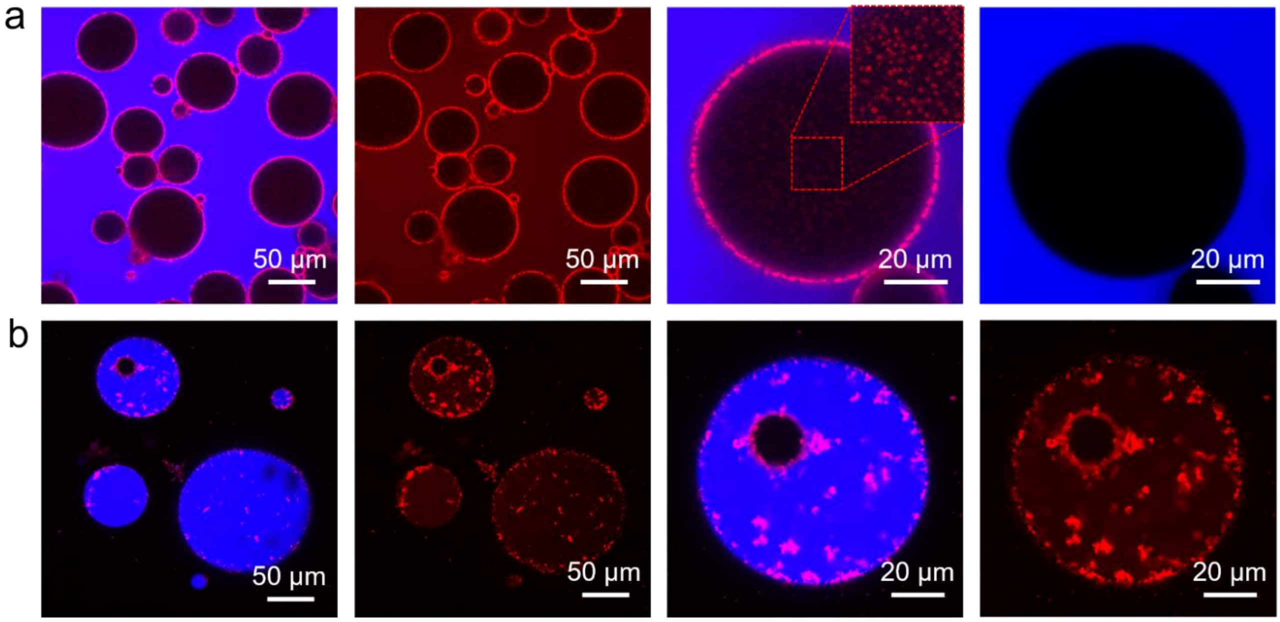

Fig. S15 CLSM images of Pickering emulsions stabilized by 0.5 wt% PNIPAM-*co*-MAA microgels (red) in the oil phase and 0.5 wt% PDEAEMA microgels (red) in the water phase at different pH values. (a) pH 1, (b) pH 11.

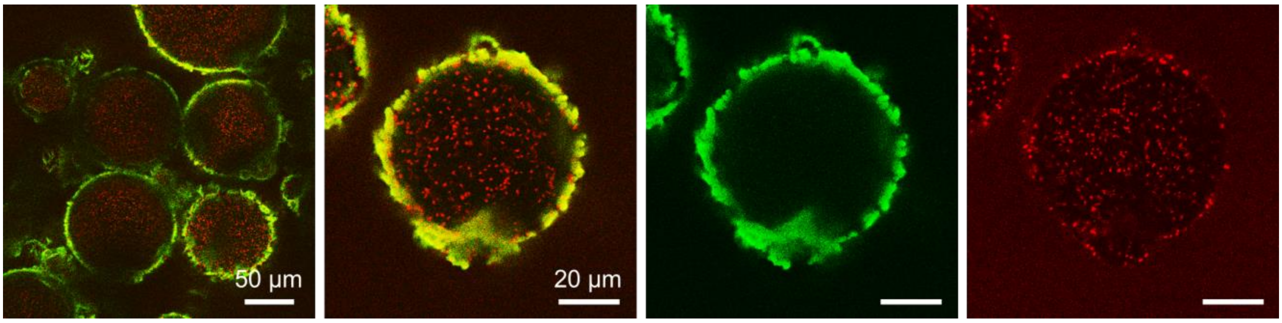

Fig. S16 CLSM images of W/O Pickering emulsions stabilized by 0.5 wt% PNIPAM-*co*-MAA microgels (green) in the oil phase, encapsulating 0.5 wt% PNIPAM-*co*-MAA microgels (red) in the water phase, respectively.

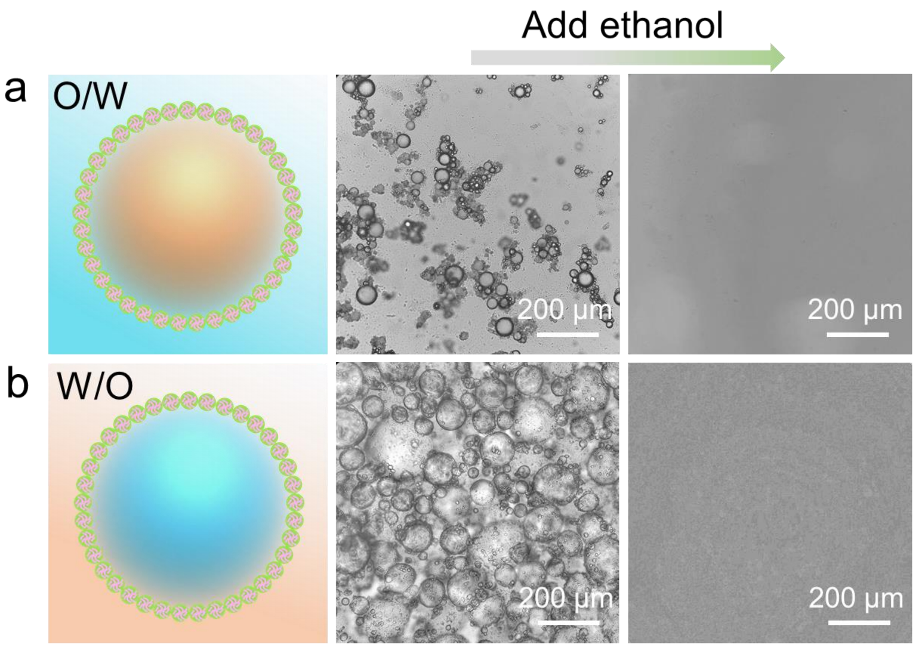

Fig. S17 Schematic illustration and optical microscopy images of (a) conventional O/W Pickering emulsion and (b) inverse W/O Pickering emulsion stabilized by 1 wt% PNIPAM-*co*-MAA microgels in water and oil phase respectively, and the demulsification of emulsions induced by additional ethanol.

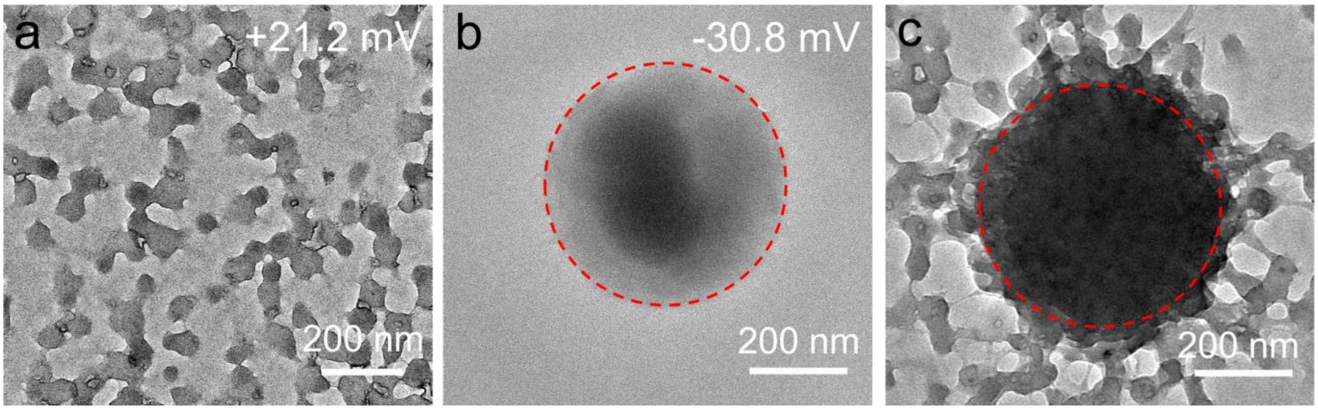

Fig. S18 Microstructure of PNIPAM-*co*-4VP microgel, PNIPAM-*co*-MAA microgel and corresponding complex particle consists of larger PNIPAM-*co*-MAA microgels as the core and smaller PNIPAM-*co*-4VP microgels as the corona.

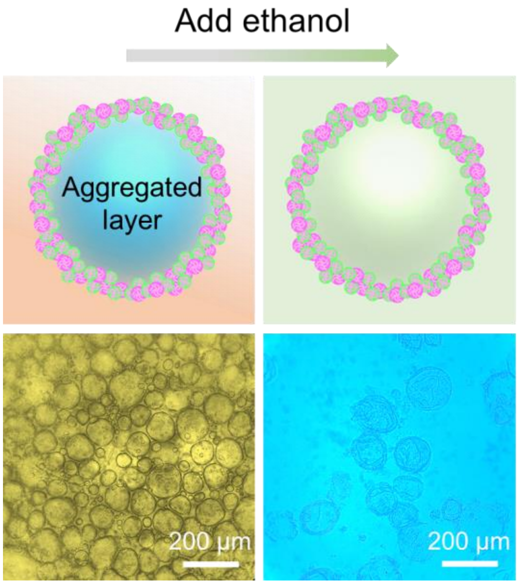

Fig. S19 Schematic illustration and morphology of microgelsomes with aggregated layer composed of oppositely charged PNIPAM-*co*-MAA microgels and PNIPAM-*co*-4VP microgels in toluene and ethanol.

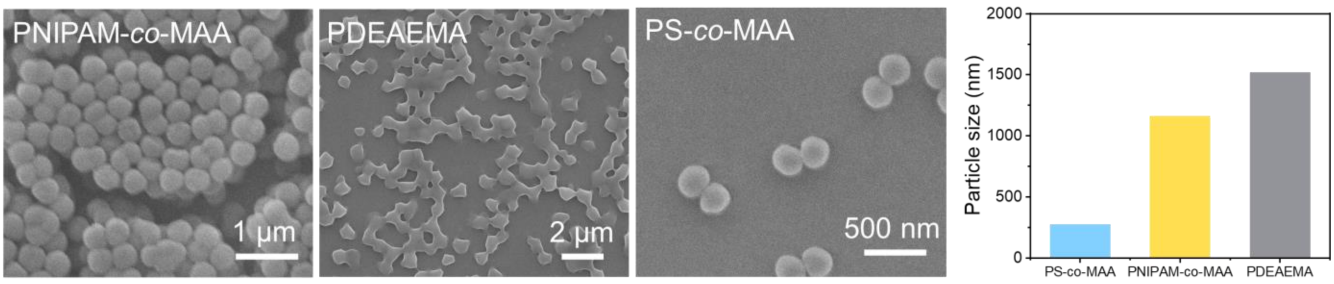

Fig. S20 Microstructure and hydrodynamic diameter of varies microgels and PS latex particles.

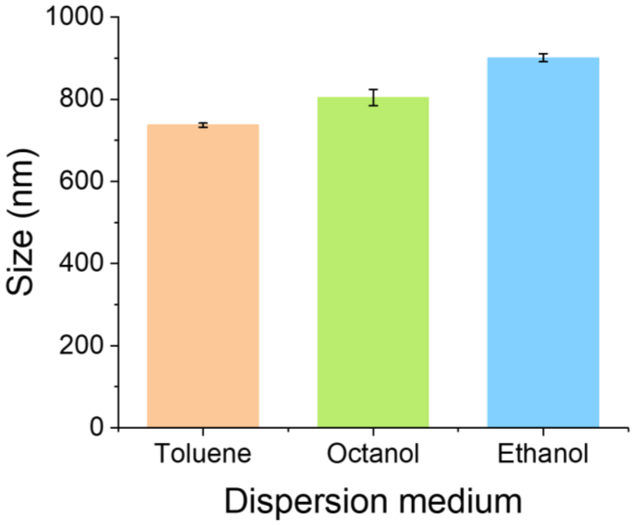

Fig. S21 Mean particle size of PNIPAM-*co*-MAA microgels in different dispersion mediums.

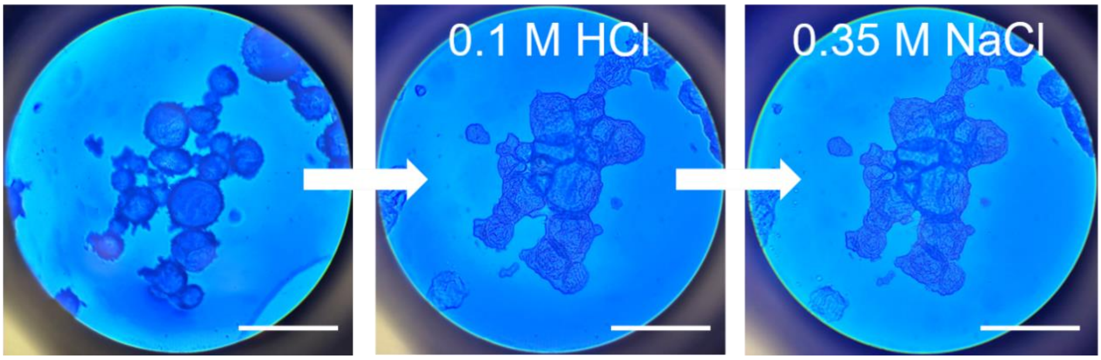

Fig. S22 The pH and salt response of microgelsomes with particle bilayer consisted of PNIPAM-*co*-MAA microgels and PDEAEMA microgels in the solutions containing different concentrations of HCl and NaCl.

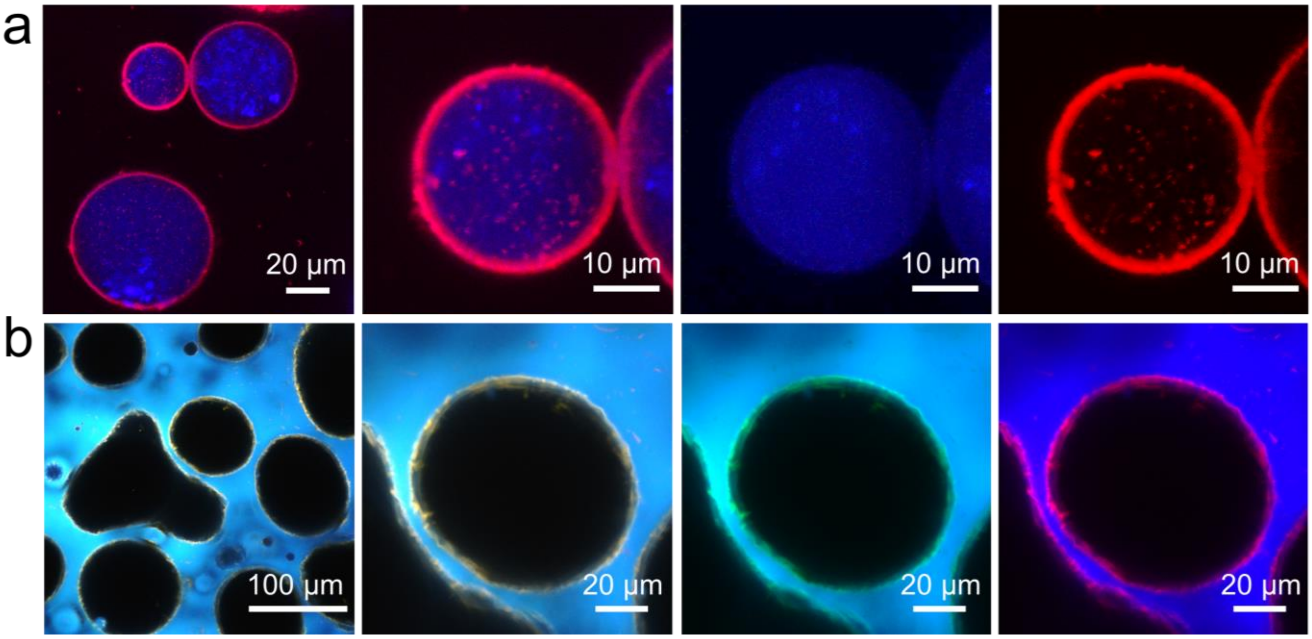

Fig. S23 W/O Pickering emulsions stabilized by negativly charged 0.5 wt% PNIPAM-*co*-MAA microgels (red) in the oil phase, containing (a) 0.5 wt% Calcofluor white stained starch nanocrystals (blue) with negative charges, and (b) 0.5 wt% FITC stained lipase (green) with positively charged residues (e.g., amino groups) on the surface in water solution.

References

1. P. Eastman, M. S. Friedrichs, J. D. Chodera, R. J. Radmer, C. M. Bruns, J. P. Ku, K. A. Beauchamp, T. J. Lane, L.-P. Wang, D. Shukla, T. Tye, M. Houston, T. Stich, C. Klein, M. R. Shirts and V. S. Pande, *Journal of Chemical Theory and Computation*, 2013, **9**, 461-469.
2. P. Eastman and V. Pande, *Computing in Science & Engineering*, 2010, **12**, 34-39.
3. K. Vanommeslaeghe, E. Hatcher, C. Acharya, S. Kundu, S. Zhong, J. Shim, E. Darian, O. Guvench, P. Lopes, I. Vorobyov and A. D. Mackerell Jr, *Journal of Computational Chemistry*, 2010, **31**, 671-690.
4. T. Darden, D. York and L. Pedersen, *The Journal of Chemical Physics*, 1993, **98**, 10089-10092.
5. Y. K. Choi, S.-J. Park, S. Park, S. Kim, N. R. Kern, J. Lee and W. Im, *Journal of Chemical Theory and Computation*, 2021, **17**, 2431-2443.
6. L. Martínez, R. Andrade, E. G. Birgin and J. M. Martínez, *Journal of Computational Chemistry*, 2009, **30**, 2157-2164.
